# Exploring the Mitogenomes of Acroneuriinae: The First Report of Gene Rearrangements in Plecoptera Species and Phylogenetic Analyses of Perlidae

**DOI:** 10.1002/ece3.72309

**Published:** 2025-10-08

**Authors:** Ying Wang, Yannan Niu, Baoni Qin, Jinjun Cao, Weihai Li, Dávid Murányi

**Affiliations:** ^1^ Henan International Joint Laboratory of Taxonomy and Systematic Evolution of Insecta Henan Institute of Science and Technology Xinxiang Henan China; ^2^ Department of Zoology Eszterházy Károly Catholic University Eger Hungary

**Keywords:** gene rearrangement, mitochondrial genome, phylogeny, stonefly

## Abstract

Perlidae represents one of the most diverse and ecologically important groups within the order Plecoptera. However, the phylogenetic relationships within Perlidae remain unresolved due to morphological plasticity and the limited availability of molecular data. Here we sequenced and assembled the mitogenome of *Hemacroneuria ovalis* and *Hesperoperla pacifica* . By comparing with other published Acroneuriinae mitogenomes, we found that minimal length variation in protein‐coding genes (PCGs), transfer RNA genes (tRNAs), and ribosomal RNA genes (rRNAs), whereas the control region (CR) exhibits considerable length divergence. Meanwhile, the mitogenome of Acroneuriinae species is relatively conserved in nucleotide composition and codon usage. The nucleotide diversity (Pi) and Ka/Ks values indicated that the *ND6* gene evolves at a faster rate than the *COI* gene, and all 13 PCGs are under purifying selection. We also identified gene rearrangement in these two mitogenomes, representing the first report of such events in the order Plecoptera. Phylogenetic results support the monophyly of the tribes Claasseniini, Neoperlini, and Kiotinini, as well as the subfamily Perlinae. Trees from the PCG and PCG12 datasets exhibited more congruent and well accepted topologies. Although both Acroneuriini and Perlini failed to demonstrate monophyly, the phylogenetic relationships among the five families of Perlidae were still reconstructed as (((Perlini + Neoperlini) + Claasseniini) + Kiotinini) + Acroneuriini.

## Graphical abstract

First reported gene rearrangements in plecopteran mitogenomes. The monophyly of the subfamily Acroneuriinae and the phylogenetic relationships among tribes within this subfamily are still controversial.

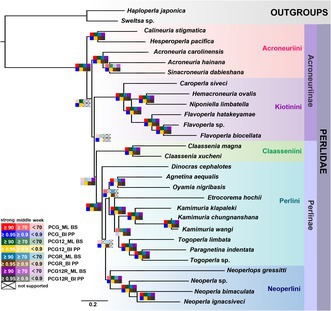

## 1 Introduction

Plecoptera, commonly known as stoneflies, are an ancient order of insects with more than 4000 species described worldwide (DeWalt et al. 2025; Zwick 2000). These insects are typically found in cold, clean, and well‐oxygenated freshwater habitats, where their larvae are aquatic and serve as sensitive bioindicators of ecosystem health (Fochetti and Tierno de Figueroa 2008). Within Plecoptera, the family Perlidae represents one of the most diverse and ecologically important groups, with over 1100 species distributed across temperate and tropical regions, particularly in North America and Asia (DeWalt et al. 2025). Perlidae nymphs play critical roles in freshwater ecosystems as predators and detritivores, contributing to nutrient cycling and energy flow (Fochetti and Tierno de Figueroa 2008). Despite their ecological importance, the phylogenetic relationships within Perlidae remain unresolved due to morphological plasticity and the limited availability of molecular data. Based on morphological data, members of Perlidae are currently assigned to two subfamilies (Acroneuriinae and Perlinae) (Zwick 2000). However, most molecular analyses recovered a paraphyletic Acroneuriinae (Mo et al. 2022; South et al. 2021; Terry 2003; Wang et al. 2022; Xiang et al. 2021). Meanwhile, phylogenetic relationships below the genera level in Perlidae are still unclear and require more molecular data.

The mitochondrial genome (mitogenome) of insects typically ranges between 14 and 20 kilobases and contains 37 conserved genes: 13 protein‐coding genes (PCGs), 22 transfer RNA (tRNA) genes, and two ribosomal RNA (rRNA) genes, along with a non‐coding control region (CR) that regulates transcription and replication (Cameron 2014). Mitogenomes are characterized by maternal inheritance, minimal recombination, rapid evolutionary rates, and abundant molecular markers (Boore 1999; Li et al. 2021; Lin et al. 2022), making them valuable tools for phylogenetic reconstruction, population genetics, and evolutionary studies (Du et al. 2019; Korkmaz et al. 2018; Miller et al. 2009; Ožana et al. 2022; Ye et al. 2018). Insect mitogenomes generally maintain conserved gene arrangements across major lineages, yet lineage‐specific rearrangements (particularly involving tRNA and protein‐coding genes) occur in certain groups and serve as phylogenetically informative markers (Boore 1999). Notably, extensive gene order rearrangements have been identified in specific groups, such as Hymenoptera, Hemiptera, Lepidoptera, and Coleoptera (Liu et al. 2017, 2019; Timmermans and Vogler 2012; Wei et al. 2010). These lineages, characterized by high species diversity, broad taxonomic representation, and distinct rearrangement patterns, are extensively studied due to their potential to elucidate evolutionary relationships and genomic plasticity (Liu et al. 2017, 2019; Timmermans and Vogler 2012; Wei et al. 2010). Aquatic insects such as Trichoptera also exhibit extensive gene order rearrangements (Ge et al. 2022, 2023). In contrast, Plecoptera remains understudied in this regard, making this order a promising candidate for exploring novel genomic evolutionary patterns.

Currently, all published stonefly mitogenomes exhibit a gene order identical to the ancestral mitogenome arrangement of insects. In this study, we sequenced the mitogenomes of two Acroneuriinae species using next‐generation sequencing technology. We analyzed the main features of the mitogenomes across subfamilies and identified novel gene rearrangement patterns, as well as their potential evolutionary processes, which were reported for the first time in Plecoptera. Furthermore, we reconstructed the phylogenetic relationships within the family Perlidae.

## 2 Materials and Methods

### 2.1 Specimen Collection and DNA Extraction

The specimens of *Hemacroneuria ovalis* were collected from Tianmu Mountain (Zhejiang Province, China), while *Hesperoperla pacifica* were obtained from the Cucharas River in Colorado, USA. Voucher specimens of *H. ovalis* (No. Vhem–0008) and *H. pacifica* (No. Vhem–0242) were deposited in the Entomological Museum of Henan Institute of Science and Technology (HIST), Henan Province, China. All specimens were preserved in 100% ethanol and maintained at −20°C until DNA extraction. Total genomic DNA was extracted from the thoracic muscle using a DNeasy Blood and Tissue Kit (Qiagen, Hilden, Germany), following the manufacturer's protocol. NanoDrop One (Thermo Scientific, USA) was used to measure the DNA concentration for each sample. DNA samples with qualified concentration (> 10 μg) were sent to Berry Genomics Co. Ltd. (Beijing, China) for further detecting.

### 2.2 Sequence Assembly, Annotation, and Analysis

The mitochondrial genome sequences of two Acroneuriinae species were obtained through high‐throughput sequencing performed by Berry Genomics Co. Ltd. (Beijing, China). For each species, DNA libraries were prepared with an average insert size of about 350 bp and then sequenced using the Illumina HiSeq 2500 platform with 150 bp paired‐end reads. Raw data were processed by trimming adapter sequences and filtering out low‐quality or short reads. High‐quality reads were then de novo assembled using IDBA‐UD (Peng et al. 2012) with k‐mer lengths ranging from 45 to 145 bp. tRNA genes and their secondary structures were predicted using the MITOS2 webserver (Donath et al. 2019) under the invertebrate mitochondrial genetic code. PCGs and rRNA genes were annotated through comparative alignment with homologous sequences from previously reported stonefly mitogenomes.

To confirm the mitogenome assemblies in the gene rearrangement areas, the target fragments were amplified by PCR using primers designed from the flanking regions of these rearranged segments. The primers for *H. pacifica* are HpaF‐CCCATTCTCCCTACGATTCT and HpaR‐ATAGAGCGTGACATTGAAGATG. The primers for *H. ovalis* are HovF‐TTGGCTCCTCATCCTTC and HovR‐GGGCTCAGGTCTTCTAAG. The target fragments were amplified and sequenced as described in our previous studies (Wang et al. 2017, 2018).

Nucleotide composition and codon usage patterns of PCGs were calculated using MEGA 7.0 (Kumar et al. 2016). Nucleotide bias was analyzed using AT‐skew [(A − T)/(A + T)] and GC‐skew [(G − C)/(G + C)] formulae, as described by Perna and Kocher (1995). Analyses of nucleotide diversity (Pi), non‐synonymous substitutions (Ka), and synonymous substitution (Ks) for the 13 PCGs were conducted employing DnaSP v5 (Librado and Rozas 2009). The gene arrangement patterns were analyzed using the CREx tool (Bernt et al. 2007) on the Galaxy platform (https://usegalaxy.eu).

### 2.3 Phylogenetic Analysis

Twenty‐five previously published perlid mitogenomes were analyzed in combination with two newly sequenced mitogenomes (Table 1) to investigate phylogenetic relationships within Perlidae. Two chloroperlid species, *Haploperla japonica* and *Sweltsa* sp. were selected as outgroups. GenBank accession numbers are listed in Table 1.

**TABLE 1:** Species used for phylogenetic analyses in this study.

Subfamily	Tribe	Species	Length	Accession number
Acroneuriinae	Acroneuriini	*Calineuria stigamatica*	15,070	MG677941^a^
		*Hesperoperla pacifica*	15,666	PV878594^a^
		*Acroneuria carolinensis*	15,718	MN969989
		*Acroneuria hainana*	15,804	NC_026104
		*Sinacroneuria dabieshana*	15,752	MK492253
	Kiotinini	*Caroperla siveci*	15,353	MG677942
		*Flavoperla hatakeyamae*	15,730	MN821010
		*Flavoperla* sp.	15,796	MN419916
		*Flavoperla biocellata*	15,805	MK905206^a^
		*Hemacroneuria ovalis*	16,351	PV878593
		*Niponiella limbatella*	15,924	MK686067
Perlinae	Claasseniini	*Claassenia magna*	15,774	MN419914
		*Claassenia xucheni*	15,746	OK012604
	Perlini	*Dinocras cephalotes*	15,666	NC_022843
		*Agnetina aequalis*	15,369	KX091846^a^
		*Oyamia nigribasis*	15,923	MN548290
		*Etrocorema hochii*	15,854	MK905888
		*Kamimuria chungnanshana*	15,943	NC_028076
		*Kamimuria klapaleki*	16,077	MN400755
		*Kamimuria wangi*	16,179	NC_024033
		*Paragnetina indentata*	15,885	MN627431
		*Togoperla* sp.	15,723	KM409708
		*Togoperla limbata*	15,915	MN969990
	Neoperlini	*Neoperla bimaculata*	15,774	OL693682
		*Neoperlops gressitti*	15,699	MN400756
		*Neoperla* sp.	15,667	KX091859^a^
		*Neoperla ignacsiveci*	15,777	KX091858
Outgroups		*Haploperla japonica*	16,012	OL351265
		*Sweltsa* sp.	15,893	OL351266

^a^ Nearly complete genome sequence.

Each PCG sequence (excluding stop codons) was individually aligned through codon‐based multiple alignment using the MAFFT algorithm on the TranslatorX platform (Abascal et al. 2010). Ambiguously aligned regions were subsequently removed with Gblocks (default parameters) prior to nucleotide conversion. For rRNA sequences, alignment was performed using the G–INS–i strategy in MAFFT 7.0 (Katoh and Standley 2013), followed by refinement via the Gblocks Server (Castresana 2000).

MEGA 7.0 (Kumar et al. 2016) was used to concatenate gene alignments into a composite matrix. Four datasets were subsequently prepared for phylogenetic analyses: PCG (all 13 PCGs), PCG12 (13 PCGs with the third codon positions removed), PCG12R (PCG12 dataset plus two rRNAs), PCGR (PCG dataset plus two rRNAs). Bayesian inference (BI) and maximum likelihood (ML) methods were used to perform phylogenetic analyses using MrBayes 3.2.6 (Ronquist et al. 2012) and IQ‐TREE (Nguyen et al. 2015), respectively. ModelFinder was used to identify the best‐fit model for each gene‐based partition of the dataset based on the Akaike Information Criterion (AIC) (Nguyen et al. 2015). The best schemes were selected and subsequently employed in BI and ML analyses (Table S1). For BI analyses, each dataset was subjected to two independent runs of 10 million generations, with trees sampled every 1000 generations and a 25% burn‐in applied. Phylogenetic trees for ML analysis were constructed using the ultrafast bootstrap approximation method with 1000 replicates. Bayesian analyses were also carried out using PhyloBayes MPI v1.4f (Lartillot et al. 2013) with the CAT + GTR model. For PhyloBayes, we ran two independent chains and stopped runs when their maxdiff value was < 0.3.

### 2.4 Phylogenetic Hypothesis Tests

To assess the phylogenetic incongruence among two conflicting nodes, a four‐clusters likelihood mapping (FcLM) analysis (Strimmer and Von Haeseler 1997) was conducted using TreePuzzle v5.3 (Schmidt et al. 2002). All four matrices were subjected to hypothesis testing under the GTR model.

## 3 Results

### 3.1 General Features of Acroneuriinae Mitogenomes

**FIGURE 1:**
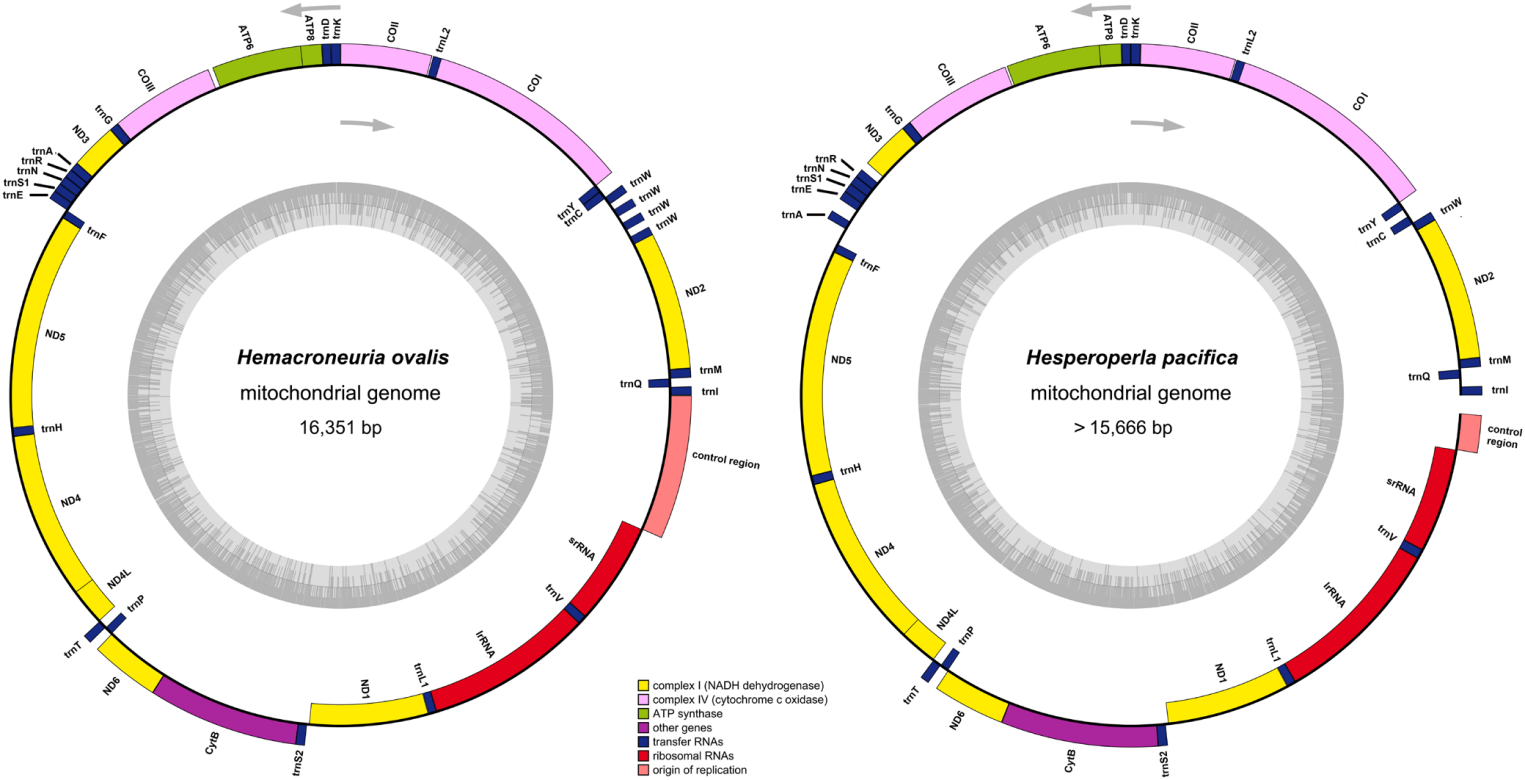
Mitochondrial maps of *Hemacroneuria ovalis* and *Hesperoperla pacifica* . Orientation of gene transcription is indicated by the arrows. PCGs are shown as blue arrows, rRNA genes as purple arrows, tRNA genes as red arrows and CR as gray arrows. tRNA genes are labeled according to single‐letter IUPAC‐IUB abbreviations (L1: UUR, L2: CUN, S1: AGN, S2: UCN). The black sliding window shows GC content and GC skew, and they are plotted as the deviation from the average value of the entire sequence.

Mitogenomes of *H. ovalis* (16,351 bp) and *H. pacifica* (15,666 bp) were sequenced and assembled (Table 1). The first sequence was complete in length, whereas the second was missing a portion of the CR (Figure 1). Comparative analysis found that the lengths of PCGs and RNAs exhibited low variance, whereas the CRs showed significant variability in length (Table S2).

**FIGURE 2:**
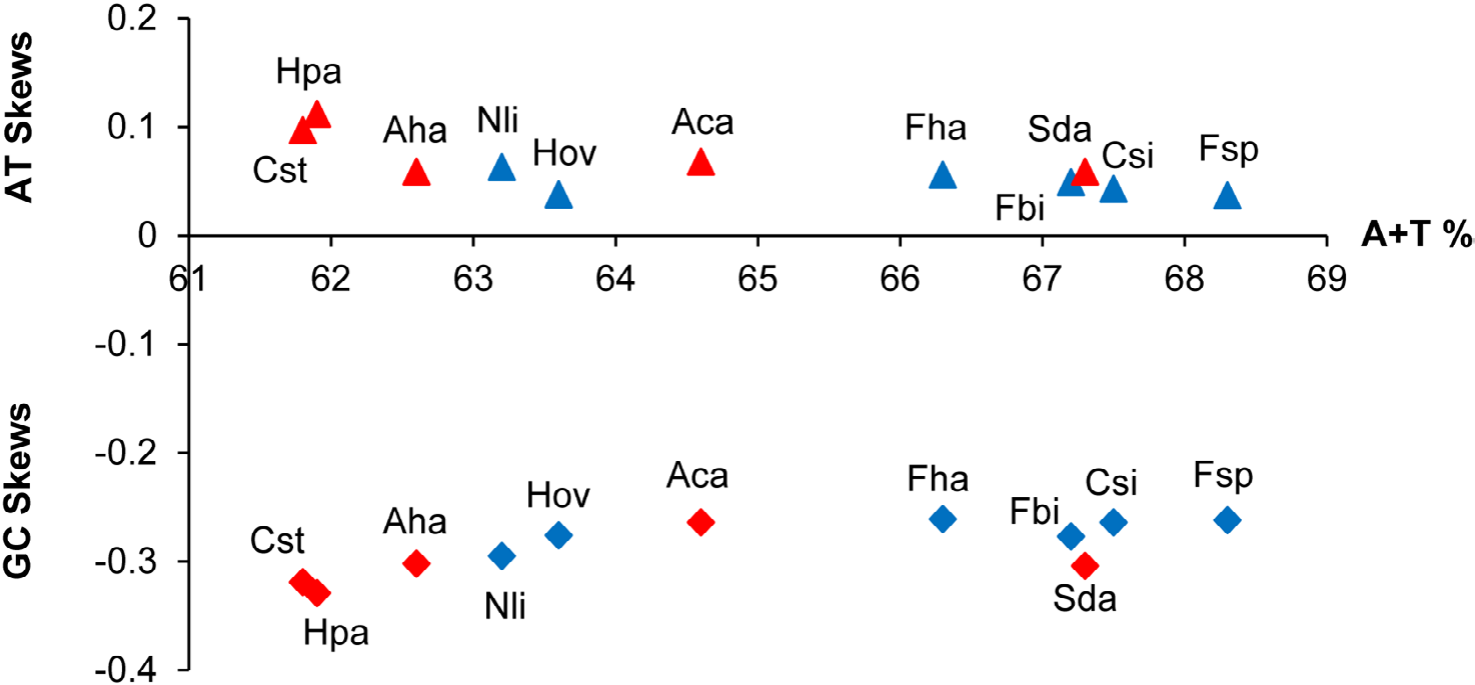
The AT‐skew, GC‐skew and A + T content in 11 Acroneuriinae mitogenomes. Red and blue represent mitogenomes from the Acroneuriini and Kiotinini, respectively. Abbreviations of species names are listed in TableS2.

The base composition patterns of *H. ovalis* and *H. pacifica* are similar, indicating a strong bias toward A and T nucleotides, with total A + T contents of 63.6% and 61.9%, respectively (Table S2). Similar to other Acroneuriinae mitogenomes, the CR has the highest A + T content in *H. ovalis* (69.2%). Furthermore, all Acroneuriinae mitogenomes exhibit positive AT‐skews and negative GC‐skews (Figure 2 and Table S2). This indicates that the mitogenomes include a greater proportion of A and C than T and G. Overall, the base composition of the Acroneuriinae mitogenomes exhibits the typical features of plecopteran mitogenomes (Mo et al. 2022; Wang et al. 2017, 2018).

Similar to the PCGs of other published stonefly species (Mo et al. 2022; Xiang et al. 2021; Wang et al. 2017, 2018), the majority of PCGs in these two species also initiated with a standard ATN codon (Tables S3 and S4). While GTG was used for *ND5* in *H. pacifica* , TTG was also proposed as the initiation codon for *ND1* in the two recently sequenced species (Tables S3 and S4). This phenomenon was commonly observed in Plecoptera and invertebrate mitogenomes, where TTG, GTG, and GTT could also serve as initiation codons for PCGs (Negrisolo et al. 2011). Most PCGs in *H. ovalis* and *H. pacifica* terminated with the typical TAA/TAG stop codons, except for the *COI* and *ND5* genes that used the incomplete stop codon T (Tables S3 and S4). This type of codon was likely a product of selective pressure to economize the mitogenome size (Rand 1993). The incomplete stop codon was relatively common in insect mitogenomes and was presumed to be corrected by post‐transcriptional polyadenylation (Ojala et al. 1981). The relative synonymous codon usage (RSCU) values of two Acroneuriinae mitogenomes were calculated (Table S5). The three most frequently used codons are UUU, UUA, and AUU, and all of them are composed of only A and/or U (Table S5).

**FIGURE 3:**
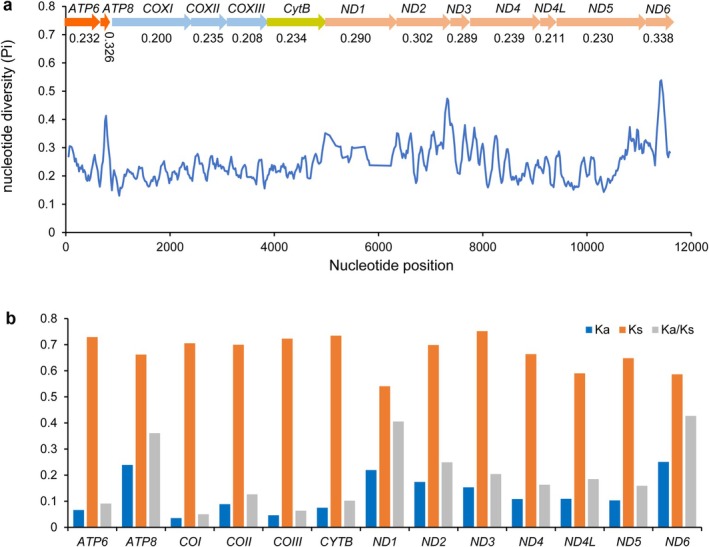
Nucleotide diversity (Pi) and Ka/Ks rates of 13 PCGs in 11 Acroneuriinae mitogenomes. (a) The blue line shows the value of nucleotide diversity Pi (window size = 100 bp, step size = 20 bp). The Pi value for each gene is shown in the graph. (b) Ka/Ks rates of 13 PCGs were calculated among the 11 Acroneuriinae species.

The nucleotide diversity (Pi) and Ka/Ks values of the 13 PCGs among 11 Acroneuriinae species were calculated (Figure 3). The Pi values ranged from 0.200 for *COI* to 0.338 for *ND6* (Figure 3a). These results suggest that *ND6* is the most divergent gene in Acroneuriinae, while *COI* is the most conserved. The Ka/Ks ratios for 13 PCGs varied from 0.050 (*COI*) to 0.427 (*ND6*) (Figure 3b), indicating the *ND6* gene evolves at a faster rate than the *COI* gene. Meanwhile, the Ka/Ks values of 13 PCGs were less than 1.0, indicating that they are under purifying selection (Hurst 2002; Yang and Bielawski 2000).

### 3.2 Gene Rearrangement of Acroneuriinae

**FIGURE 4:**
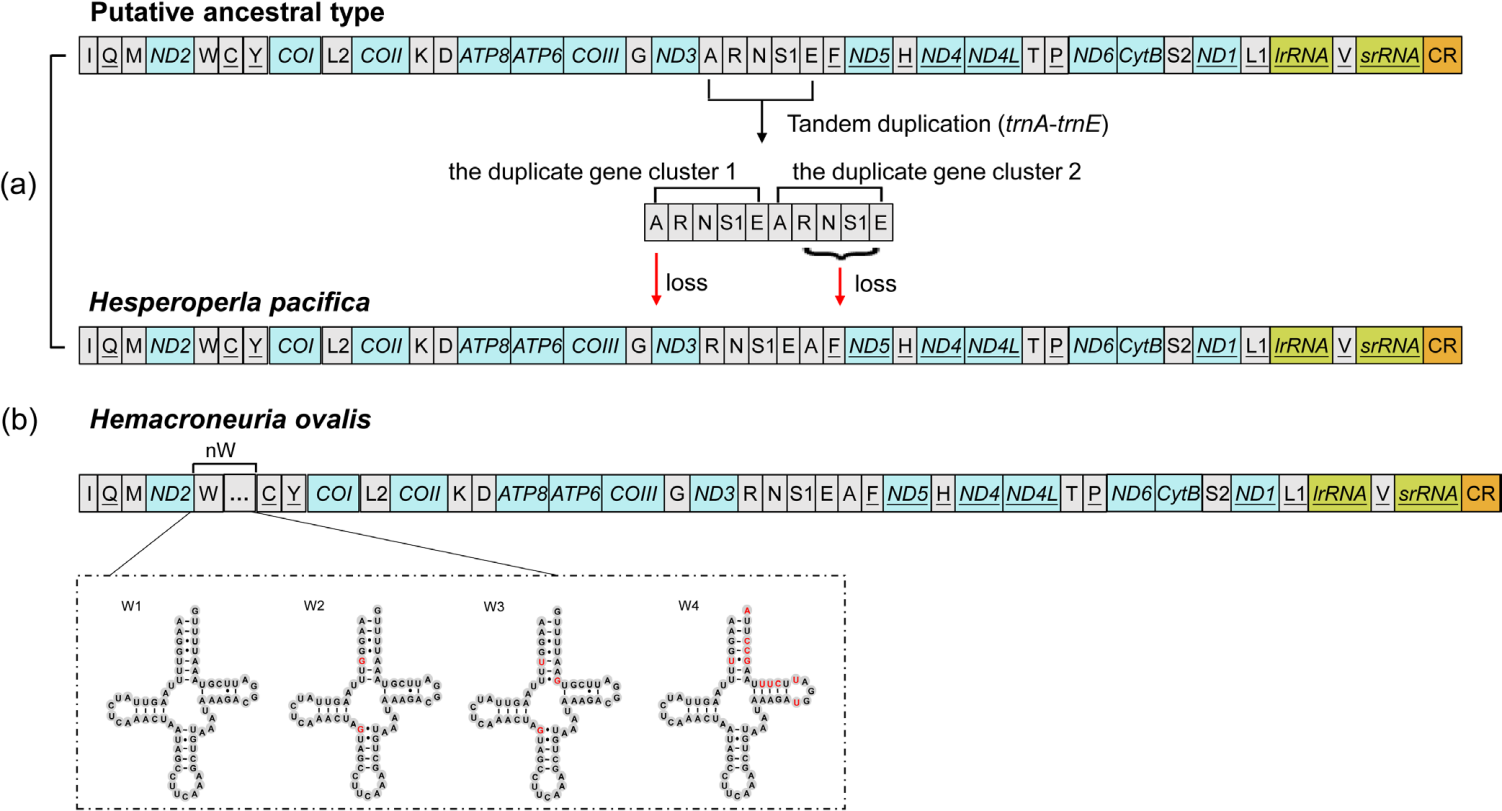
Putative gene rearrangement events in *Hesperoperla pacifica* (a) and *Hemacroneuria ovalis* (b) mitogenome. Gene names that are not underlined indicate a forward transcriptional direction, whereas underlines indicate a reverse transcriptional direction. The black arrow indicates gene duplication. The red arrow indicates absence of a gene. Red circles represent nucleotides not conserved in four predicted *trnW* genes.

In this study, gene rearrangements were found in two newly sequenced mitogenomes (Figure 4), which are the first to be identified in stonefly mitogenomes. Both species exhibit distinct patterns of tRNA rearrangement. In the *H. pacifica* mitogenome, there is a gene rearrangement changing the ancestral gene order of *trnA*‐*trnR*‐*trnN*‐*trnS1*‐*trnE* to the novel gene order *trnR*‐*trnN*‐*trnS1*‐*trnE*‐*trnA* (Figure 4a). In contrast, an unusual duplication of the *trnW* gene was observed in the *H. ovalis* mitogenome, resulting in four copies located between the *ND2* and *trnC* genes (Figure 4b).

### 3.3 Phylogenetic Relationships Within Perlidae

**FIGURE 5:**
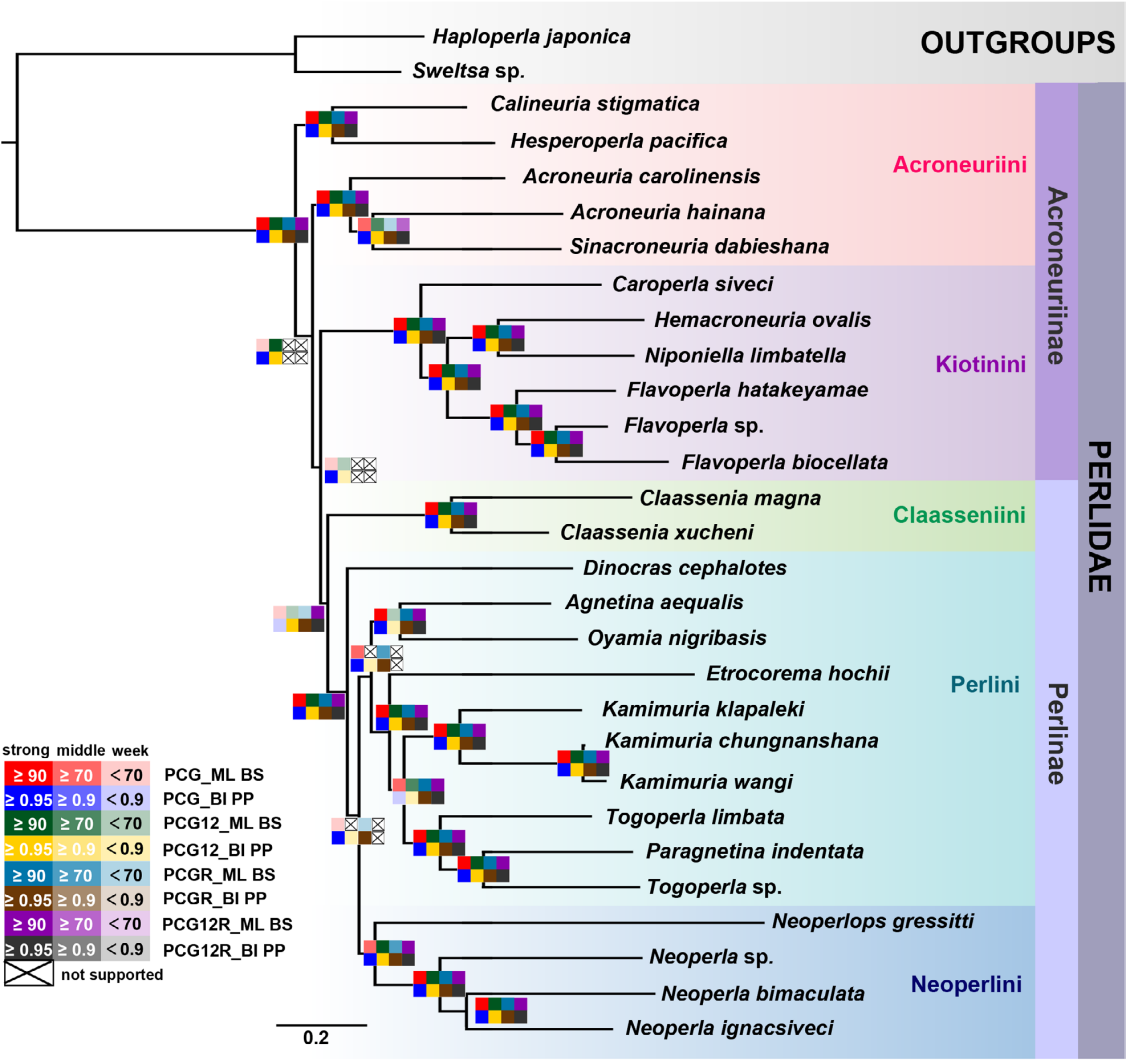
Phylogeny of Perlidae inferred from the ML tree based on the PCG dataset. Squares with different colors at nodes represent the support rates from different datasets and analytical methods as shown at the bottom left. Values at nodes represent the Bayesian posterior probabilities (PPs) or bootstrap probabilities (BSs).

Four datasets (PCG, PCG12, PCG12R, and PCGR) were used for phylogenetic analyses under BI and ML, which produced four different topologies (Figure 5 and Figures S1–S4). All trees supported the monophyly of Perlinae, along with that of most tribes. The topologies from the PCG dataset were nearly identical to those derived from the PCG12 dataset, except for the inconsistent positioning of the clade containing *Agnetina aequalis* and *Oyamia nigribasis* (Figures S1 and S2). Although both Acroneuriini and Perlini failed to demonstrate monophyly, the phylogenetic relationships among the five families of Perlidae were still reconstructed as (((Perlini + Neoperlini) + Claasseniini) + Kiotinini) + Acroneuriini (Figures S1 and S2).

Phylogenetic analyses using BI and ML conducted separately on the PCGR and PCG12R datasets both yielded congruent topologies (Figures S3 and S4). However, the inconsistency between two topologies was localized to the phylogenetic placement of the *A. aequalis* + *O. nigrigasis* clade. In addition, all trees consistently revealed the nesting of Kiotinini within Acroneuriini, failing to recover the monophyly of Acroneuriini (Figures S3 and S4).

**FIGURE 6:**
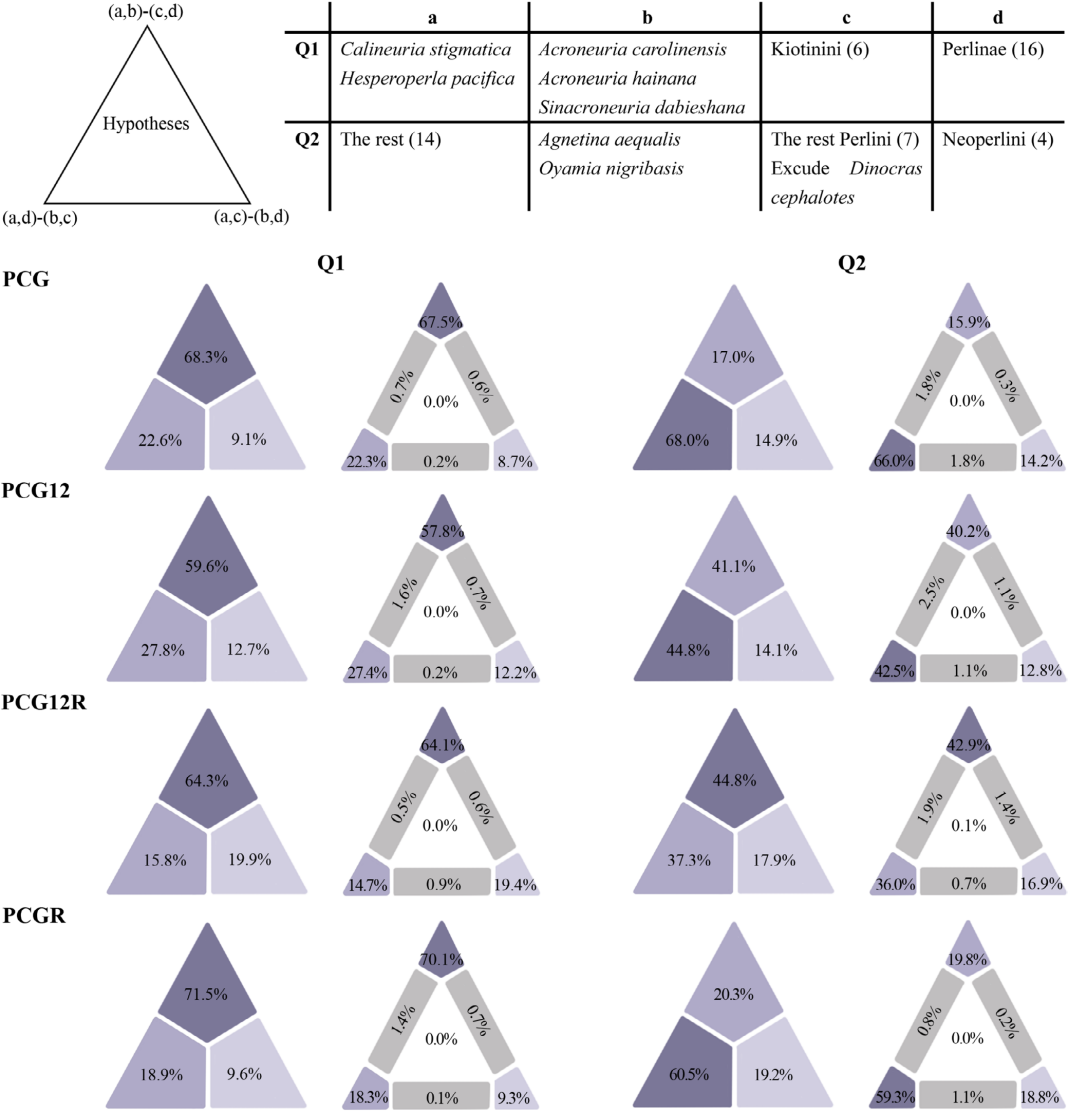
Results from four‐clusters likelihood mapping showing support for the hypotheses of conflict nodes. The clusters of tested conflicts Q1 and Q2 are displayed in the included table. The analysis was based on four datasets, PCG, PCG12, PCG12R, and PCGR. The deeper colors represent the higher support values for the three regions, which indicate different hypotheses for the relationship of four clusters (a–d). The number of species is indicated for corresponding groups in brackets in the included table.

Phylogenetic incongruence was assessed through four‐clusters likelihood mapping (FcLM) to test two key topological conflicts: (1) whether Kiotinini or the clade ( *Acroneuria carolinensis* + (*A. hainana* + *Sinacroneuria dabieshana*)) represents the sister group to Perlinae; and (2) the relationships among the clade ( *A. aequalis* + *O. nigribasis*), the remaining seven Perlini species, and Neoperlini. Analyses of four datasets (PCG, PCG12, PCG12R, PCGR) revealed moderate support for Kiotinini as the sister to Perlinae (68.3%/59.6%/64.3%/71.5%). The remaining conflicts were unresolved, though FcLM analyses of PCG, PCG12, and PCGR datasets favored a sister‐group relationship between ( *A. aequalis* + *O. nigribasis*) and the remaining Perlini (68.0%/44.8%/60.5%), while PCG12R weakly supported an alternative topology grouping Neoperlini with the remaining Perlini (44.8%) (Figure 6).

## 4 Discussion

### 4.1 Mitogenome Features of Acroneuriinae

In the present study, two Acroneuriinae mitogenomes are sequenced and assembled, of which *H. ovalis* is a complete mitogenome and *H. pacifica* is a linear mitogenome. The size of *H. ovalis* mitogenome is the longest among known Acroneuriinae mitogenomes (Table S2). Comparative analyses reveal minimal length variation in PCGs, tRNAs, and rRNAs, whereas the CR exhibits considerable length divergence (Table S2). Like other published plecopteran mitogenomes (Mo et al. 2022; Xiang et al. 2021; Wang et al. 2017, 2018), Acroneuriinae mitogenomes exhibit A + T‐rich nucleotide composition, with positive AT‐skew and negative GC‐skew (Table S2). In two newly sequenced mitogenomes, most PCGs employ ATN start codons (ATA, ATT, ATG) and terminate with TAA or TAG stop codons (Tables S3 and S4). The codons terminating with A or U show a clear preference over both four‐fold and two‐fold degenerate codons (Table S5). Furthermore, the three most frequently used codons are all composed of A and/or T (Table S5). This phenomenon is commonly observed in other published Acroneuriinae and plecopteran species, indicating a conservation in codon usage. Overall, the mitogenome of Acroneuriinae species is relatively conserved in nucleotide composition and codon usage.

### 4.2 Gene Rearrangements in Plecopteran Mitogenomes

Mitochondrial gene order in insects is generally conserved; however, rearrangements do occur during evolution in certain lineages (Boore 1999). Mitochondrial gene rearrangements have now been documented across numerous insect orders; however, they exhibit significant variation in both frequency and scale. Within orders exhibiting frequent rearrangements (e.g., Phthiraptera, Psocoptera, Thysanoptera, and Hymenoptera), rearrangements have been detected in all examined taxa and often involve numerous genes, including PCGs, rRNA genes, and tRNA genes (Dowton and Austin 1999; Dowton et al. 2002). Notably, within Phthiraptera, Psocoptera, and Thysanoptera, rearrangements occur not only in recognized rearrangement hotspots but also within regions typically considered highly conserved (Schmidt and Barker 2001). However, orders exhibiting fewer rearrangements (e.g., Hemiptera, Diptera, and Coleoptera) generally show rearrangements in only a limited number of species and involve fewer genes. In some orders (e.g., Lepidoptera), all species share identical rearrangement patterns, suggesting these rearrangements may represent synapomorphies for specific lineages (Cao et al. 2012). Given that mitochondrial gene rearrangement events in insects contain valuable genetic information relevant to species phylogeny and evolution (Cameron 2014), more thorough and systematic investigation of rearrangements across all insect orders is warranted. To date, all known plecopteran mitogenomes exhibit conserved ancestral gene arrangements. In this study, we identified two cases of gene rearrangement in the subfamily Acroneuriinae, representing the first report of such events in the order Plecoptera. Given that mitogenomic gene order remains largely conserved and ancestral across Plecoptera, we infer that the origin and evolution of these rearrangements in Acroneuriinae likely represent independent events, at least at the subfamily level within Plecoptera.

Multiple mechanisms have been proposed to explain mitochondrial gene rearrangements in insects. Among these hypotheses, the tandem duplication‐random loss (TDRL) model is generally accepted as the most common mechanism for such rearrangements in this taxonomic group (Boore 2000). This model suggests that slipped‐strand mispairing or erroneous termination during replication causes partial regions of the mitogenome to be duplicated, and the random deletion of supernumerary gene copies results in the emergence of novel gene orders (Lavrov et al. 2002; Mauro et al. 2006). In addition, other mechanisms like inversion (Smith et al. 1989), tandem duplication/nonrandom loss (TDNL) (Lavrov et al. 2002), tRNA duplication/anticodon mutation (Cantatore et al. 1987; Rawlings et al. 2003), and recombination (Lunt and Hyman 1997) have also been proposed to account for rearrangement events that cannot be fully explained by the TDRL model.

tRNA genes generally exhibit the highest degree of mobility, although different tRNAs move at distinct rates (Moritz and Brown 1987). While tRNAs located between PCGs are often considered relatively stable, specific clusters containing *trnA*–*trnR*–*trnN*–*trnS1*–*trnE*–*trnF* or *trnI*–*trnQ*–*trnM* are known to undergo rearrangements most readily within Insecta (Moreno‐Carmona et al. 2021; Song et al. 2016). In this study, the gene rearrangement events of *H. pacifica* mitogenome predicted by CREx suggest that these rearrangements are likely caused by the TDRL model. It is likely that the translocation of *trnA* resulted from a tandem duplication of the gene cluster *trnA*–*trnR*–*trnN*–*trnS1*–*trnE*, which produced the *trnA*–*trnR*–*trnN*–*trnS1*–*trnE–trnA*–*trnR*–*trnN*–*trnS1*–*trnE* arrangement. The first copy of *trnA* and the second copy of *trnR*–*trnN*–*trnS1*–*trnE* were then randomly lost. This left the *trnR*–*trnN*–*trnS1*–*trnE*–*trnA* arrangement (Figure 4a). The *H. pacifica* mitogenome exhibits a gene rearrangement pattern identical to that of *Agapetus zniachtl* (Trichoptera; Ge et al. 2023).

However, an unusual duplication of the *trnW* gene was observed in *H. ovalis* mitogenome. Between the *ND2* and *trnC* genes, we identified four complete *trnW* sequences (Figure 4b). Each *trnW* is separated by 55 identical base pairs (Table S3), and the sequence of these 55 bases is identical to the sequence at the 3ʹ end of the *ND2* gene. This specific feature can be explained by the TDRL model, which ultimately generated four *trnW* copies, bringing the total number of tRNA genes in the mitogenome to 24. This phenomenon is relatively rare in insect mitogenomes, although more than 22 tRNAs have been observed in other species, such as *Brontostoma colossus* (Heteroptera; Kocher et al. 2014), *Phalantus geniculatus* (Hemiptera; Sun et al. 2019), *Reduvius tenebrosus* (Hemiptera; Jiang et al. 2016), and *Theopropus elegans* (Mantodea; Liu et al. 2023). The four *trnW* gene copies exhibit high sequence identity (> 94.49%), indicating they likely originated from a recent duplication event. In addition, all four *trnW* have a secondary structure of standard mitochondrial tRNA genes (Kumazawa and Nishida 1993; Kumazawa et al. 2014) and had an identical anticodon sequence (Figure 4b), so we speculate that all of them are functional genes.

Gene rearrangements were found to occur only in two Acroneuriinae species, involving the gene clusters *trnA* to *trnE* and/or *trnW*. This phenomenon warrants further investigation. Mitochondrial gene rearrangement has been associated with life‐history traits and high metabolic demands (Castro et al. 2002; Dowton and Campbell 2001; Moreno‐Carmona et al. 2021). A constant flow of well‐oxygenated water enhances respiration and supports an increased metabolic rate (Mackay and Wiggins 1979). Given that perlid species spend most of their life cycle (eggs and nymphs) in such aquatic environments, their mitochondrial architecture is likely strongly influenced by the conditions or lifestyle of one or more immature stages. Future studies still require more extensive investigations and comprehensive sampling to elucidate the evolution of gene rearrangements in Perlidae and Plecoptera mitogenomes.

### 4.3 Implications for the Phylogeny

We reconstructed phylogenetic relationships among Perlidae using BI and ML methods based on different datasets of mitogenomes (Figure 5). The sampling range did not cover all extant tribes of Perlidae, as mitogenomes for representatives of Anacroneuriini have not yet been sequenced. In this study, BI and ML analyses based on the same dataset generated similar topologies, with the exception of the PCG12 dataset (Figures S1–S4). However, analyses based on different datasets produced conflicting nodes. Notably, phylogenies reconstructed from the PCG and PCG12 datasets exhibited more congruent and well‐accepted topologies. Within the subfamily Perlinae, incorporation of RNA genes elevated nodal support values in Bayesian analyses. Nevertheless, this approach also inferred two controversial nodes, such as the monophyly of the tribe Acroneuriini and the position of *A. aequalis* plus *O. nigribasis* clade. Despite this limitation, datasets including and excluding RNA genes yielded complementary insights for phylogenetic inference.

The monophyly of two subfamilies, Perlinae and Acroneuriinae, is widely accepted and supported by morphological data (Zwick 2000). However, molecular evidence has consistently failed to strongly support this, particularly the monophyly of Acroneuriinae (Mo et al. 2022; South et al. 2021; Terry 2003; Wang et al. 2022; Xiang et al. 2021). In this study, all the BI and ML trees supported Perlinae as a monophyletic group (Figure 5) and confirmed the sister‐group relationship between Claasseniini and the clade comprising Perlini and Neoperlini, as previously proposed by Sivec et al. (1988). But unfortunately, the monophyly of Acroneuriinae remained unsupported (Figure 5). Notably, all topologies supported that Perlini is a paraphyletic group, which is consistent with other molecular findings based on mitogenomic and transcriptomic data (Mo et al. 2022; South et al. 2021; Wang et al. 2022; Xiang et al. 2021).

Our analyses based on the PCG and PCG12 datasets support the view that Kiotinini is closer to Perlinae, which is congruent with the conclusions of Xiang et al. (2021), but contradicts those of Wang et al. (2022) and Mo et al. (2022). However, results from the PCGR and PCG12R datasets grouped three Acroneurlini species with Perlinae in a clade, which was then sister to Kiotinini. A recent transcriptomic study also revealed a non‐monophyletic Acroneuriinae, with Kiotinini nested within Acroneuriini (South et al. 2021). We also employed FcLM analysis to solve these conflicting relationships (Figure 6); all FcLM results support the sister‐group relationship between Kiotinini and Perlinae, as recovered in the phylogenetic trees based on PCG and PCG12 datasets. Within Perlinae, phylogenetic trees from all datasets failed to support the monophyly of Perlini, and the placement of the *A. aequalis* + *O. nigribasis* clade was inconsistent. However, FcLM analyses demonstrated that three of the four datasets (excluding PCGR) consistently supported the *A. aequalis* + *O. nigribasis* clade as sister to other Perlini species (Figure 6).

Overall, our phylogenetic results support the monophyly of the tribes Claasseniini, Neoperlini, and Kiotinini, as well as the subfamily Perlinae. However, the monophyly of the subfamily Acroneuriinae and the phylogenetic relationships among tribes within this subfamily are still controversial. The reason for this result might be that an ancient rapid divergence caused substitutional saturation; the remaining few effective signals could not fully resolve these nodes (Whitfield and Lockhart 2007), and the resulting long branch attraction (LBA) was due to low taxon sampling (Siddall and Whiting 1999). Here, we first analyzed pairwise comparisons of sequence divergency using AliGROOVE with default parameters (Kück et al. 2014) and found that all pairwise sequence comparisons were positive (Figure S5), indicating low heterogeneity. Despite the low sequence heterogeneity, we still used heterogeneous models (CAT + GTR) to reduce the effects of LBA. Using this approach, the tribes Claasseniini, Neoperlini, and Kiotinini, as well as the subfamily Perlinae, were recovered as a monophyletic group. However, the posterior probabilities on some nodes are very low (< 0.5), and relationships among tribes within Acroneuriinae are still not exactly solved (Figures S6–S9). Therefore, compared with homogeneous models, LBA had no obvious effect on the topologies estimated under heterogeneous models. Due to the relatively limited number of sequenced mitogenomes in Perlidae, particularly within the subfamily Acroneuriinae, we have yet to reach a definitive conclusion regarding its phylogenetic relationships. Further studies are needed to validate these disputed phylogenetic relationships by dense taxonomic sampling and improved inference methods.

## Author Contributions

**Ying Wang:** conceptualization (equal), data curation (equal), formal analysis (equal), methodology (equal), validation (equal), writing – original draft (equal), writing – review and editing (equal). **Yannan Niu:** data curation (equal), investigation (equal). **Baoni Qin:** formal analysis (equal), software (equal). **Jinjun Cao:** funding acquisition (equal), project administration (equal), validation (equal), writing – original draft (equal), writing – review and editing (equal). **Weihai Li:** investigation (equal). **Dávid Murányi:** investigation (equal).

## Ethics Statement

No additional permission was required for the collection of samples used in this study; we confirm that their collection complied with local laws, regulations, and ethical standards. The authors have nothing to report.

## Conflicts of Interest

The authors declare no conflicts of interest.

## Supporting information


**Figure S1:** The congruent topology from the analysis of ML‐PCG (BSs in left), BI‐PCG (PPs in middle), and BI‐PCG12 (PPs in right). Values at node represented the Bayesian posterior probabilities (PPs) or bootstrap probabilities (BSs).
**Figure S2:** The topology from the analysis of ML‐PCG12. Values at node represented the bootstrap probabilities (BSs).
**Figure S3:** The congruent topology from the analysis of ML‐PCG12R (BSs in left), and BI‐PCG12R (PPs in right). Values at node represented the Bayesian posterior probabilities (PPs) or bootstrap probabilities (BSs).
**Figure S4:** The congruent topology from the analysis of ML‐PCGR (BSs in left), and BI‐PCGR (PPs in right). Values at node represented the Bayesian posterior probabilities (PPs) or bootstrap probabilities (BSs).
**Figure S5:** Heterogeneous sequence divergence within Perlidae mitochondrial genomes. The mean similarity score between sequences is represented by a colored square, based on AliGROOVE scores ranging from −1, indicating great difference in rates from the remainder of the data set, that is, heterogeneity (red coloring), to +1, indicating rates match all other comparisons (blue coloring).
**Figure S6:** BI tree based on PCG dataset with heterogeneous models (CAT+GTR). Values at node represented the Bayesian posterior probabilities (PPs).
**Figure S7:** BI tree based on PCG12 dataset with heterogeneous models (CAT+GTR). Values at node represented the Bayesian posterior probabilities (PPs).
**Figure S8:** BI tree based on PCG12R dataset with heterogeneous models (CAT+GTR). Values at node represented the Bayesian posterior probabilities (PPs).
**Figure S9:** BI tree based on PCGR dataset with heterogeneous models (CAT+GTR). Values at node represented the Bayesian posterior probabilities (PPs).
**Table S1:** Best partitioning scheme and model selected by ModelFinder for phylogenetic analyses.
**Table S2:**. Nucleotide composition of mitochondrial genomes of the 11 Acroneuriinae species.
**Table S3:** Features of the mitochondrial genome of *Hemacroneuria ovalis*.
**Table S4:** Features of the mitochondrial genome of *Hesperoperla pacifica* .
**Table S5:** Codon number and Relative synonymous codon usage (RSCU) in the *Hemacroneuria ovalis* (left) and *Hesperoperla pacifica* (right) mitochondrial PCGs.

## Data Availability

The annotated mitogenomic sequences of *Hemacroneuria ovalis* and *Hesperoperla pacifica* have been deposited in GenBank (https://www.ncbi.nlm.nih.gov/) under the accession numbers PV878593–PV878594.
