# Supplementary material for: Exploring the Mitogenomes of Acroneuriinae: The First Report of Gene Rearrangements in Plecoptera Species and Phylogenetic Analyses of Perlidae
Source: Ecol Evol. 2025 Oct 8;15(10):e72309. doi: 10.1002/ece3.72309 (PMC12508255; doi:10.1002/ece3.72309)
Supplement: Supplementary file 1 — Figure S1: The congruent topology from the analysis of ML‐PCG (BSs in left), BI‐PCG (PPs in middle), and BI‐PCG12 (PPs in right). Values at node represented the Bayesian posterior probabilities (PPs) or bootstrap probabilities (BSs). Figure S2: The topology from the analysis of ML‐PCG12. Values at node represented the bootstrap probabilities (BSs). Figure S3: The congruent topology from the analysis of ML‐PCG12R (BSs in left), and BI‐PCG12R (PPs in right). Values at node represented the Bayesian posterior probabilities (PPs) or bootstrap probabilities (BSs). Figure S4: The congruent topology from the analysis of ML‐PCGR (BSs in left), and BI‐PCGR (PPs in right). Values at node represented the Bayesian posterior probabilities (PPs) or bootstrap probabilities (BSs). Figure S5: Heterogeneous sequence divergence within Perlidae mitochondrial genomes. The mean similarity score between sequences is represented by a colored square, based on AliGROOVE scores ranging from −1, indicating great difference in rates from the remainder of the data set, that is, heterogeneity (red coloring), to +1, indicating rates match all other comparisons (blue coloring). Figure S6: BI tree based on PCG dataset with heterogeneous models (CAT+GTR). Values at node represented the Bayesian posterior probabilities (PPs). Figure S7: BI tree based on PCG12 dataset with heterogeneous models (CAT+GTR). Values at node represented the Bayesian posterior probabilities (PPs). Figure S8: BI tree based on PCG12R dataset with heterogeneous models (CAT+GTR). Values at node represented the Bayesian posterior probabilities (PPs). Figure S9: BI tree based on PCGR dataset with heterogeneous models (CAT+GTR). Values at node represented the Bayesian posterior probabilities (PPs). Table S1: Best partitioning scheme and model selected by ModelFinder for phylogenetic analyses. Table S2:. Nucleotide composition of mitochondrial genomes of the 11 Acroneuriinae species. Table S3: Features of the mitochondrial genome of H [file ECE3-15-e72309-s001.docx]

Supplementary data

**
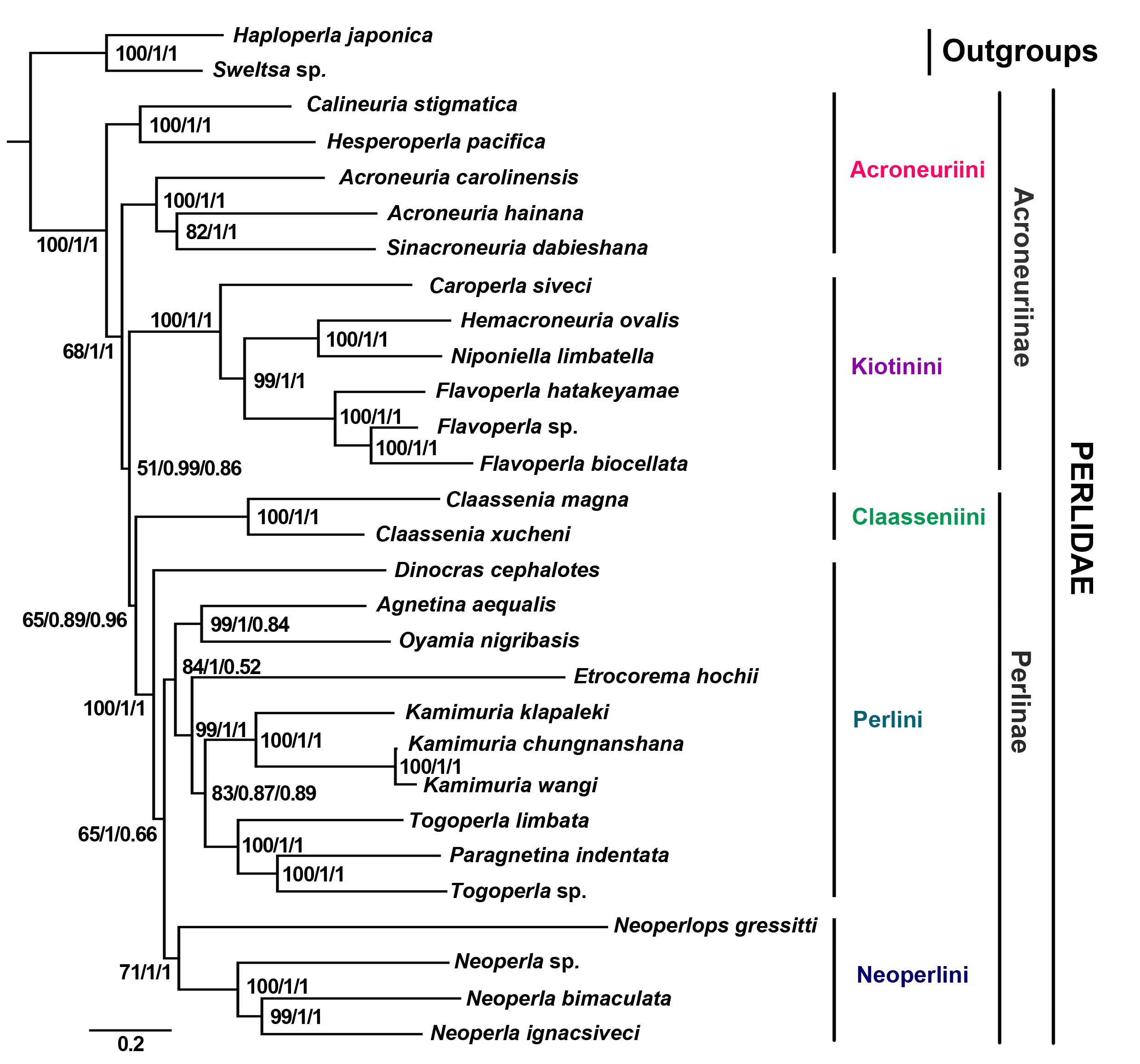
**

**Figure S1.** The congruent topology from the analysis of ML-PCG (BSs in left), BI-PCG (PPs in middle), and BI-PCG12 (PPs in right). Values at node represented the Bayesian posterior probabilities (PPs) or bootstrap probabilities (BSs).


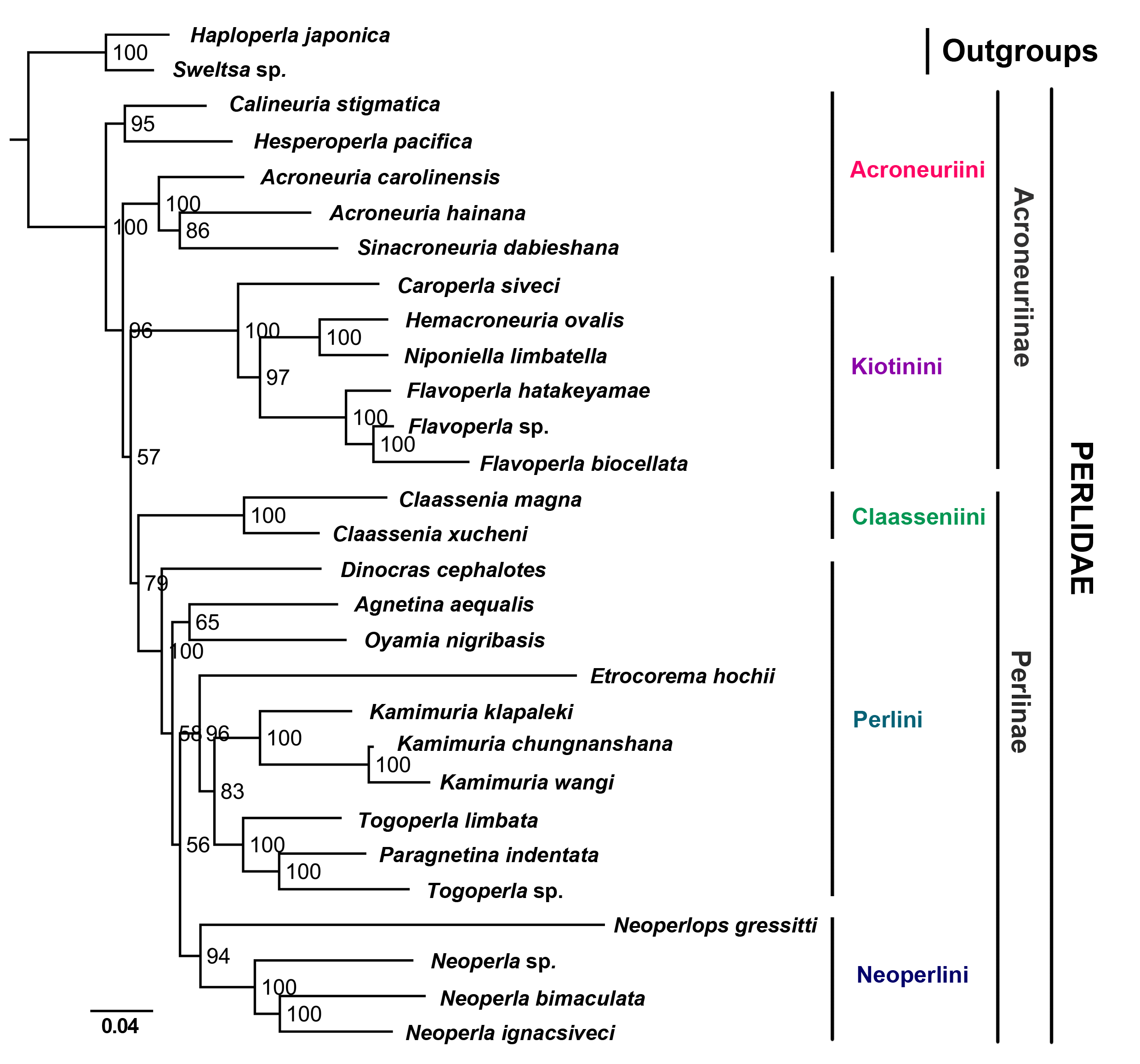


**Figure S2.** The topology from the analysis of ML-PCG12. Values at node represented the bootstrap probabilities (BSs).


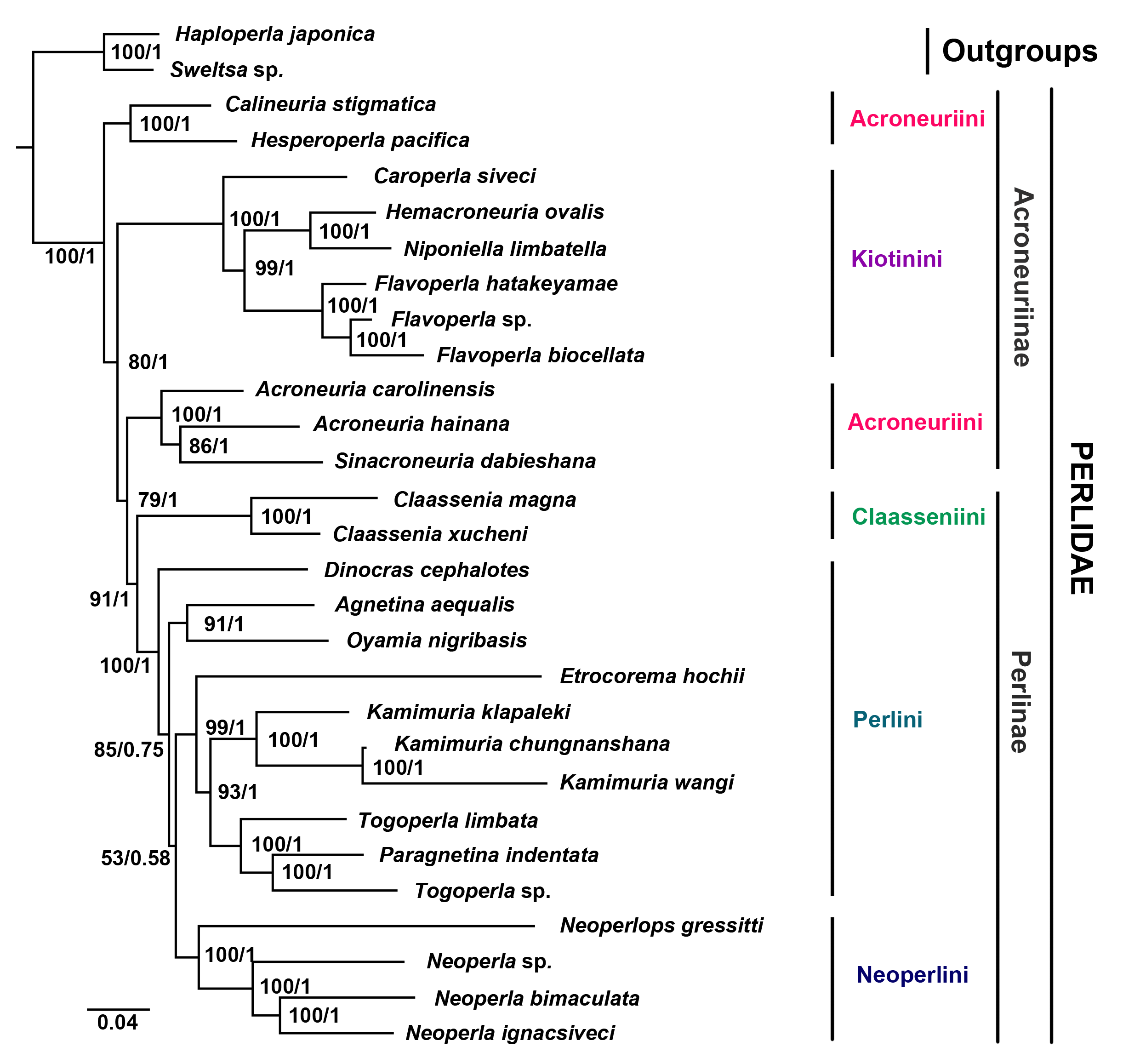


**Figure S3.** The congruent topology from the analysis of ML-PCG12R (BSs in left), and BI-PCG12R (PPs in right). Values at node represented the Bayesian posterior probabilities (PPs) or bootstrap probabilities (BSs).


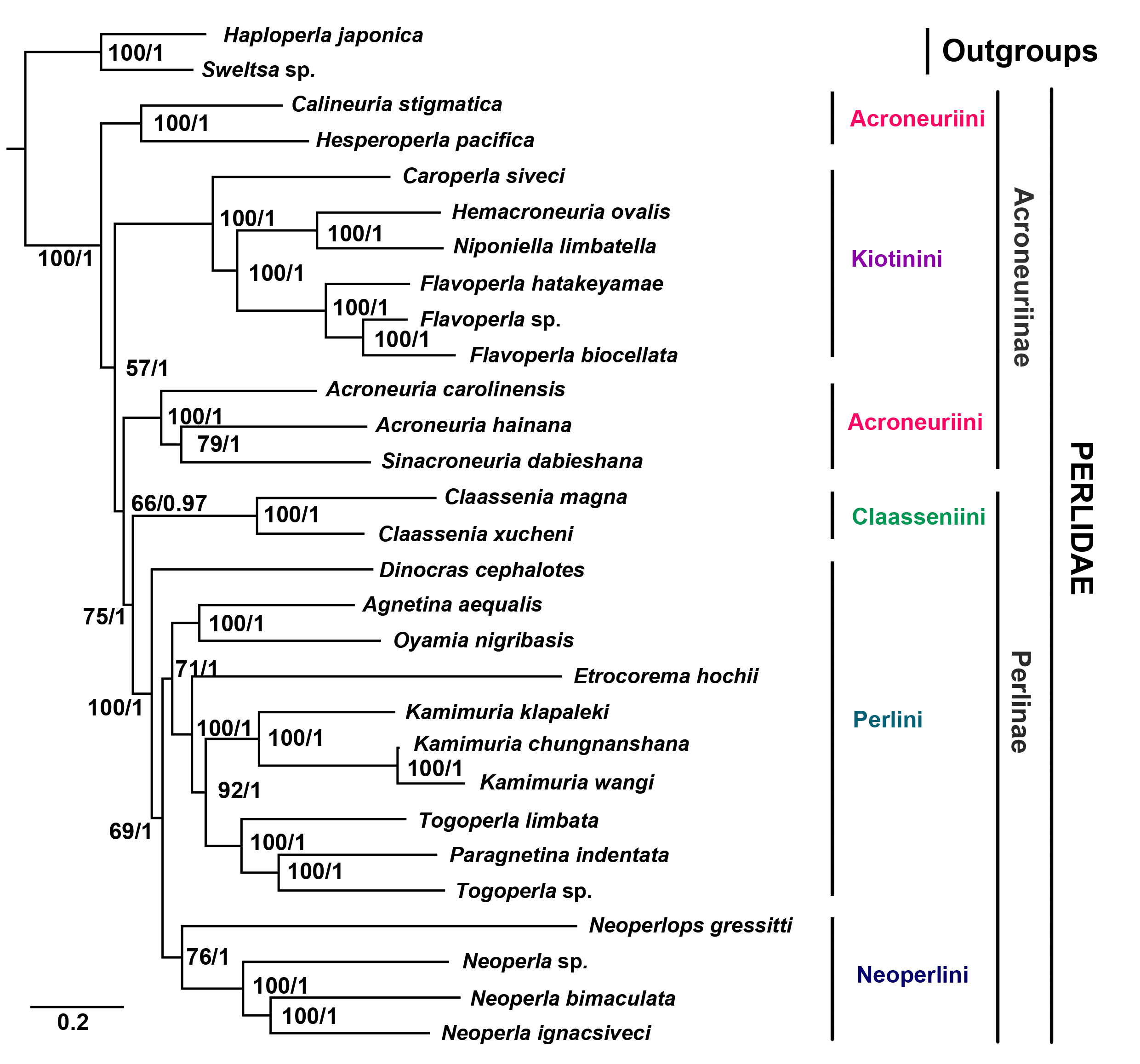


**Figure S4.** The congruent topology from the analysis of ML-PCGR (BSs in left), and BI-PCGR (PPs in right). Values at node represented the Bayesian posterior probabilities (PPs) or bootstrap probabilities (BSs).


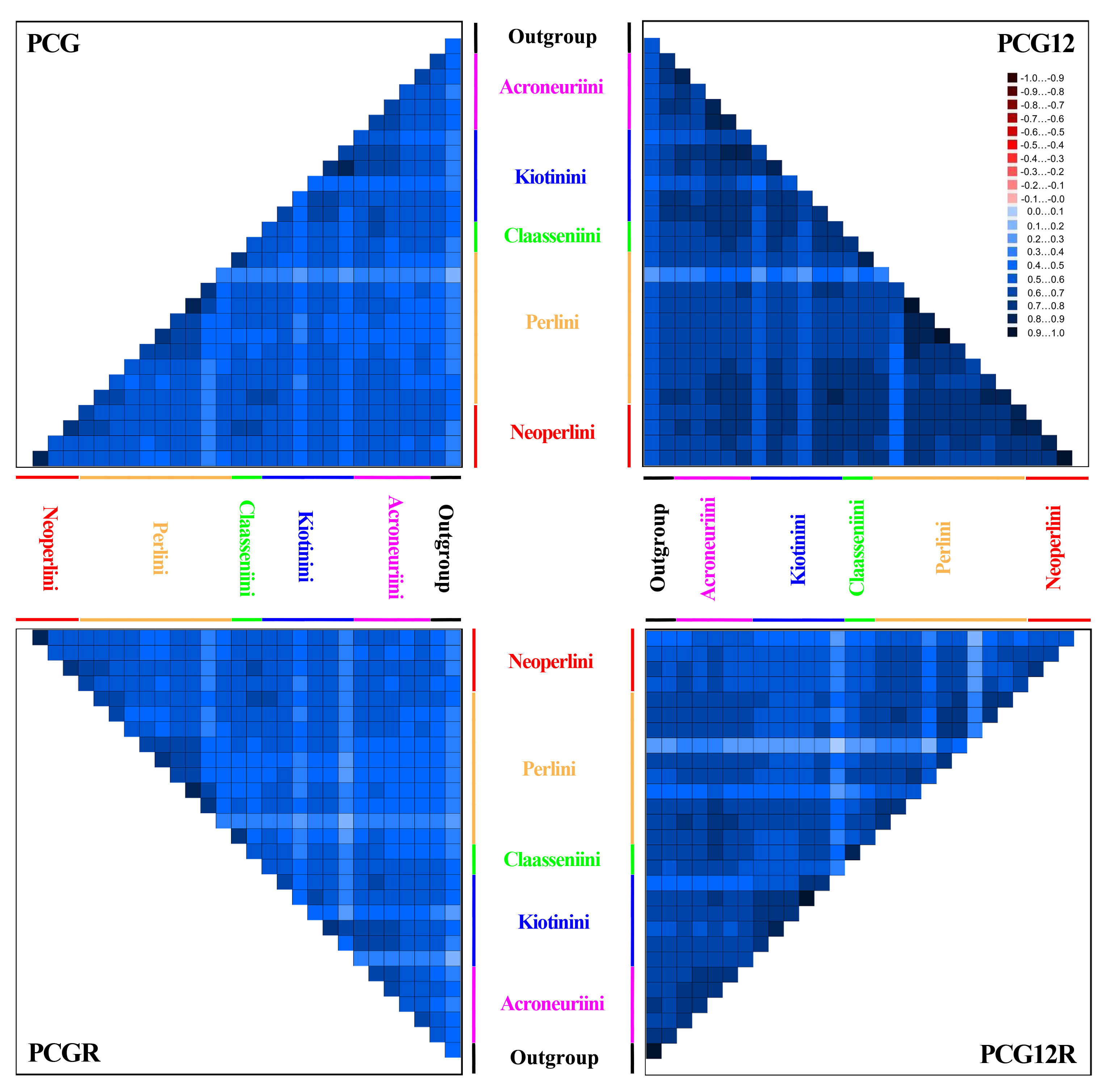
 **Figure S5.** Heterogeneous sequence divergence within Perlidae mitochondrial genomes. The mean similarity score between sequences is represented by a colored square, based on AliGROOVE scores ranging from −1, indicating great difference in rates from the remainder of the data set, i.e. heterogeneity (red coloring), to +1, indicating rates match all other comparisons (blue coloring).


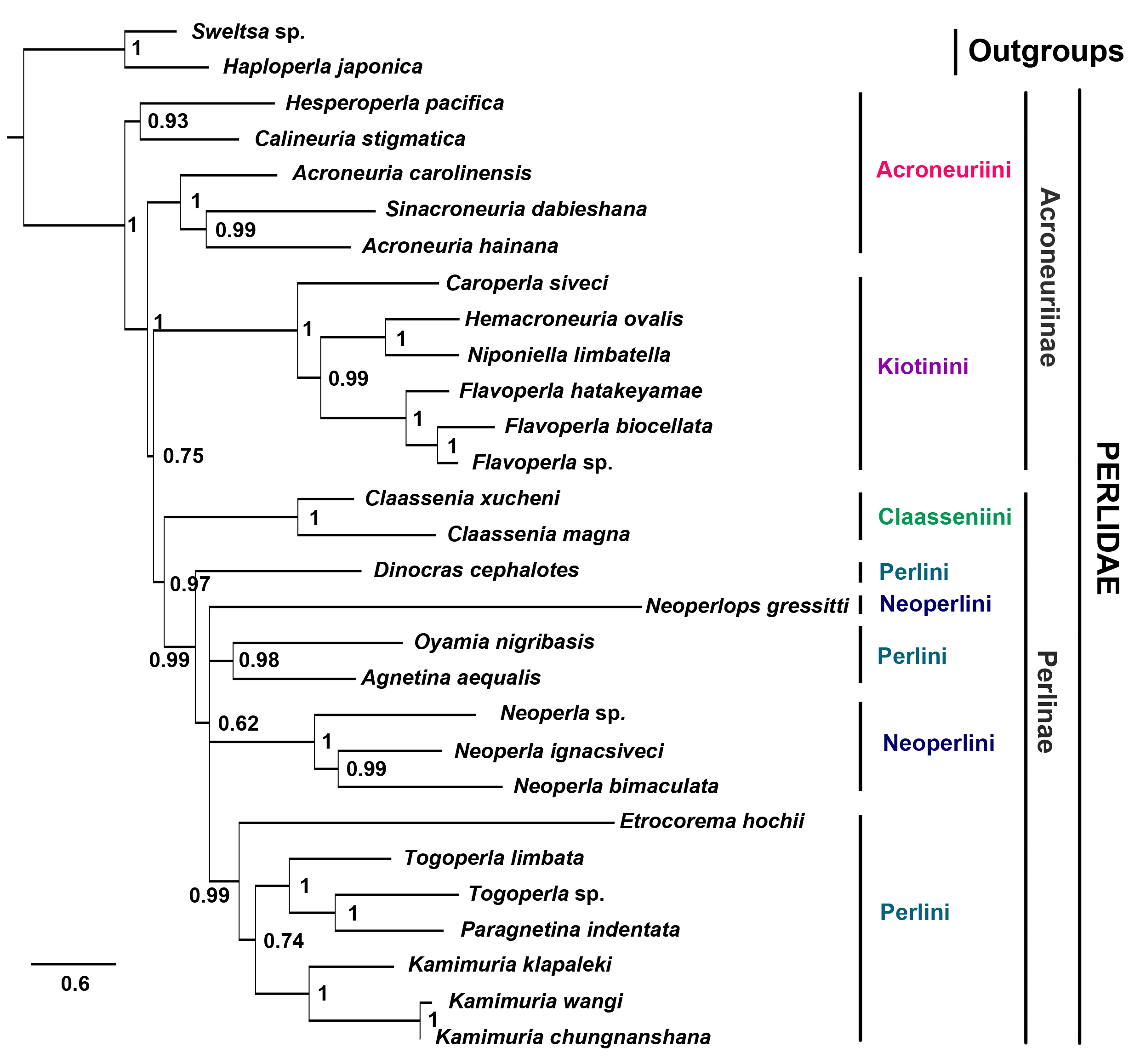


**Figure S6.** BI tree based on PCG dataset with heterogeneous models (CAT+GTR). Values at node represented the Bayesian posterior probabilities (PPs).


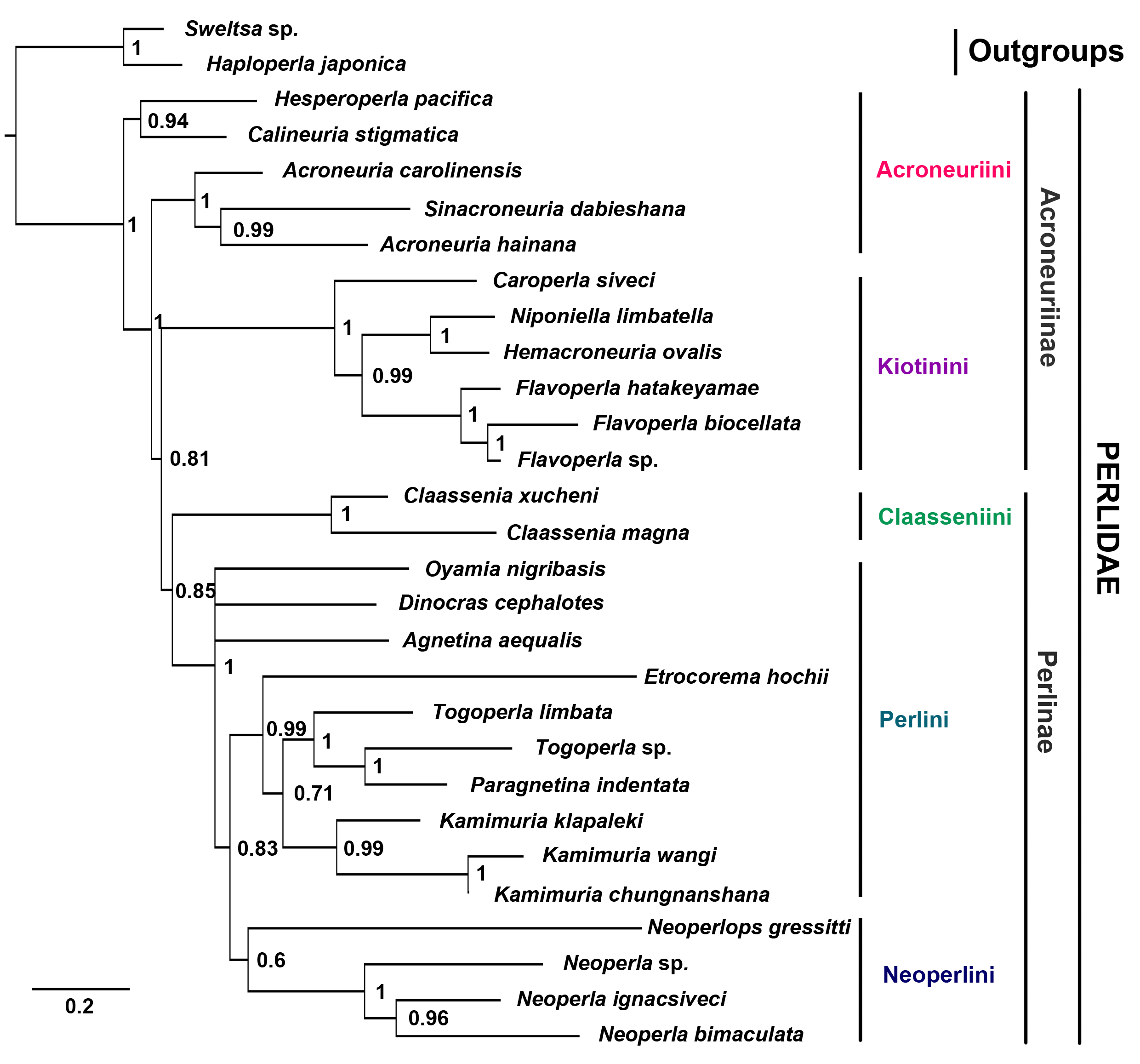


**Figure S7.** BI tree based on PCG12 dataset with heterogeneous models (CAT+GTR). Values at node represented the Bayesian posterior probabilities (PPs).


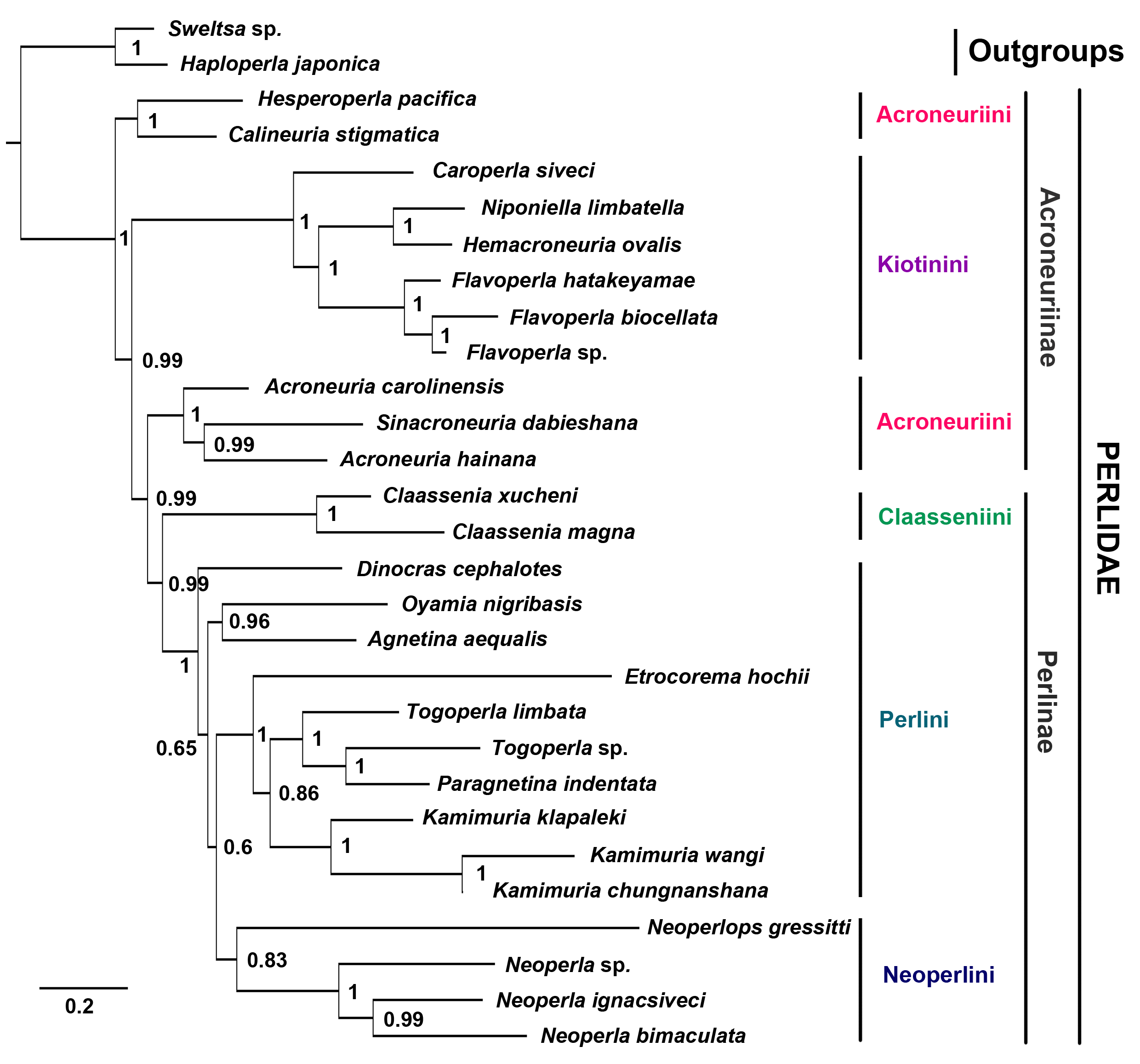


**Figure S8.** BI tree based on PCG12R dataset with heterogeneous models (CAT+GTR). Values at node represented the Bayesian posterior probabilities (PPs).


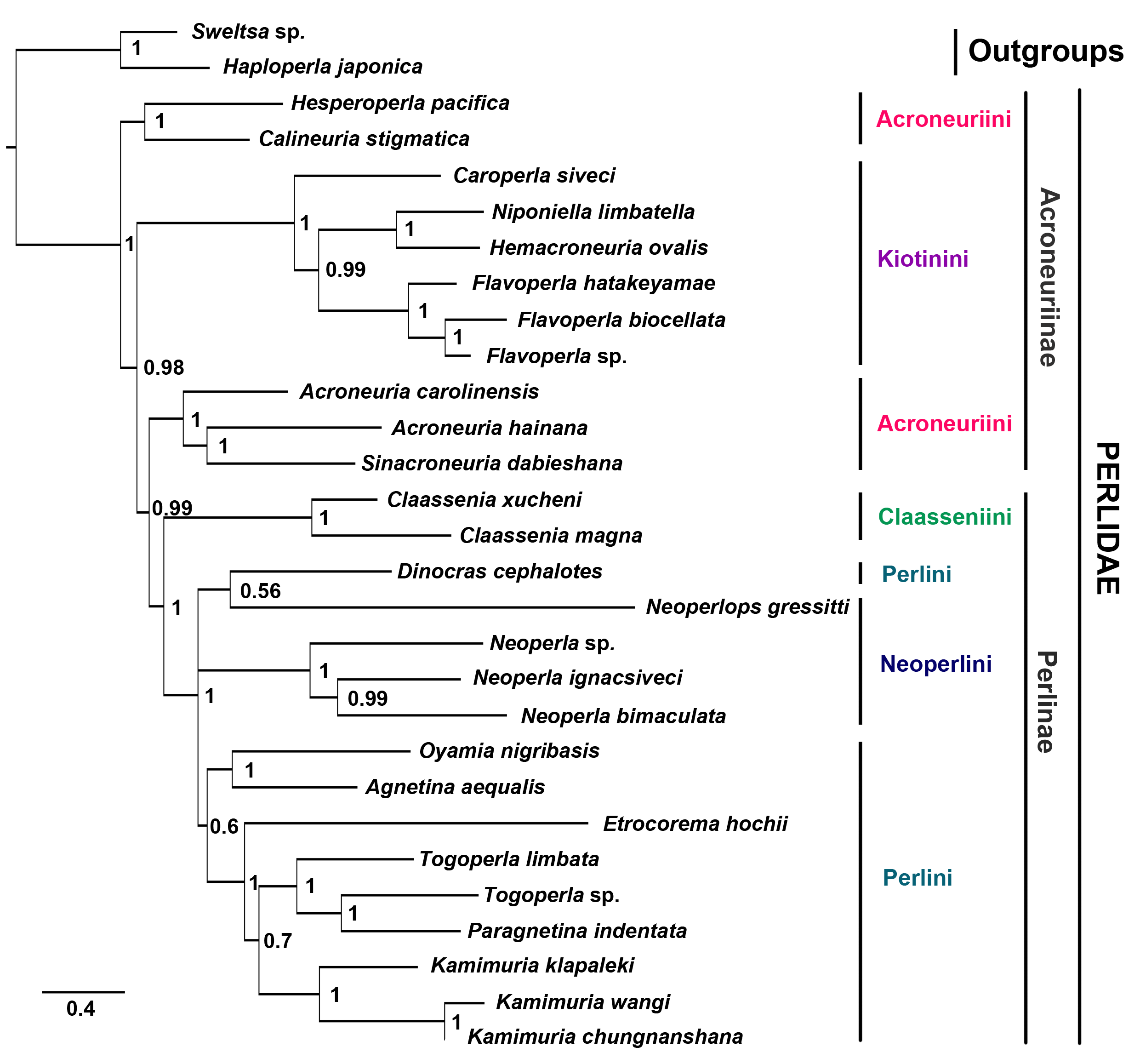


**Figure S9.** BI tree based on PCGR dataset with heterogeneous models (CAT+GTR). Values at node represented the Bayesian posterior probabilities (PPs).

**Table S1.** Best partitioning scheme and model selected by ModelFinder for phylogenetic analyses.

| Dataset and Partition type | Subset Partitions and Model for MrBayes | Subset Partitions and Model for IQ-TREE |
| --- | --- | --- |
| PCG gene partition | P1: (ATP6): GTR + I + G  P2: (ATP8): GTR + I + G  P3: (COI): GTR + I + G  P4: (COII): GTR + I + G  P5: (COIII): GTR + I + G  P6: (CYTB): GTR + I + G  P7: (ND1): GTR + G  P8: (ND2): HKY + I + G  P9: (ND3): GTR + I + G  P10: (ND4, ND5): GTR + I + G  P11: (ND4L): HKY + I + G  P12: (ND6): GTR + I + G | P1: (ATP6): TIM2 +I  P2: (ATP8): GTR + I + G  P3: (COI): GTR + I  P4: (COII): GTR  P5: (COIII): GTR + I  P6: (CYTB): GTR  P7: (ND1): GTR  P8: (ND2): HKY + I  P9: (ND3): GTR  P10: (ND4, ND5): GTR  P11: (ND4L): TN  P12: (ND6): TIM |
| PCGR gene partition | P1: (ATP6): GTR + I + G  P2: (ATP8): GTR + I + G  P3: (COI): GTR + I + G  P4: (COII): GTR + I + G  P5: (COIII): GTR +I+G  P6: (CYTB): GTR + I + G  P7: (ND1): GTR + I + G  P8: (ND2): HKY + I + G  P9: (ND3): GTR + I + G  P10: (ND4, ND5): GTR + I + G  P11: (ND4L): HKY + I + G  P12: (ND6): GTR + I + G  P13: (16s rRNA): GTR + G  P14: (12s rRNA): GTR + I + G | P1: (ATP6): TIM2 + I  P2: (ATP8): GTR  P3: (COI): GTR + I  P4: (COII): GTR  P5: (COIII): TIM2 + I  P6: (CYTB): GTR + I  P7: (ND1): GTR  P8: (ND2): TN + I  P9: (ND3): GTR  P10: (ND4, ND5): GTR  P11: (ND4L): TN  P12: (ND6): TIM  P13: (16s rRNA): GTR  P14: (12s rRNA): TIM3 + I |
| PCG12 | P1: (ATP6, CYTB): GTR + I + G  P2: (ATP8): GTR + I + G  P3: (COI): GTR + I + G  P4: (COII): GTR + I + G  P5: (COIII): GTR + I + G  P6: (ND1): GTR + G  P7: (ND2): GTR + I + G  P8: (ND3): GTR + I + G  P9: (ND4, ND5): GTR + I + G  P10: (ND4L): HKY + I + G  P11: (ND6): GTR + I + G | P1: (ATP6, CYTB): GTR + I  P2: (ATP8): GTR  P3: (COI): TIM2 + I  P4: (COII): GTR  P5: (COIII): GTR  P6: (ND1): GTR  P7: (ND2): TIM3 + I + G  P8: (ND3): GTR + I + G  P9: (ND4, ND5): TVM  P10: (ND4L): HKY  P11: (ND6): GTR |
| PCG12R | P1: (ATP6, COIII): GTR + I + G  P2: (ATP8): GTR + I + G  P3: (COI): GTR + I + G  P4: (COII): GTR + I + G  P5: (CYTB): GTR + I + G  P6: (ND1): GTR + G  P7: (ND2): GTR + I + G  P8: (ND3): GTR + I + G  P9: (ND4, ND5): GTR + I + G  P10: (ND4L): HKY + I + G  P11: (ND6): GTR + I + G  P12: (16s rRNA): GTR + G  P13: (12s rRNA): GTR + I + G | P1: (ATP6, COIII): GTR  P2: (ATP8): GTR  P3: (COI): TIM2 + I  P4: (COII): GTR  P5: (CYTB): GTR + I  P6: (ND1): GTR  P7: (ND2): TIM3 + I + G  P8: (ND3): GTR + I + G  P9: (ND4, ND5): TVM + I  P10: (ND4L): HKY  P11: (ND6): GTR  P12: (16s rRNA): GTR  P13: (12s rRNA): TIM3 + I |

**Table S2**. Nucleotide composition of mitochondrial genomes of the 11 Acroneuriinae species.

| Species |  | Whole genome | | | | | PCGs | | tRNAs | | rRNAs | | CR | |
| --- | --- | --- | --- | --- | --- | --- | --- | --- | --- | --- | --- | --- | --- | --- |
|  | Abbreviation | Length (bp) | A+T% | G+C% | AT Skew | GC Skew | Length (bp) | A+T% | Length (bp) | A+T% | Length (bp) | A+T% | Length (bp) | A+T% |
| *Acroneuria carolinensis* | Aca | 15,718 | 64.6 | 35.4 | 0.068 | -0.264 | 11,223 | 62.7 | 1,487 | 67.8 | 2,163 | 68.6 | 829 | 73.9 |
| *Acroneuria hainana* | Aha | 15,804 | 62.7 | 37.3 | 0.059 | -0.302 | 11,211 | 60.4 | 1,487 | 66.2 | 2,208 | 67.0 | 899 | 73.3 |
| *Calineuria stigmatica* | Cst | 15,070 | 61.8 | 38.2 | 0.097 | -0.319 | 11,217 | 60.2 | 1,476 | 67 | 2,112 | 67.6 | >254 | - |
| *Caroperla siveci* | Csi | 15,353 | 67.6 | 32.4 | 0.043 | -0.264 | 11,223 | 65.7 | 1,490 | 71.2 | 2,169 | 72.9 | >461 | - |
| *Flavoperla biocellata* | Fbi | 15,805 | 67.2 | 32.8 | 0.049 | -0.277 | 11,217 | 65.7 | 1,490 | 70.4 | 2,195 | 70.5 | 852 | 73.2 |
| *Flavoperla hatakeyamae* | Fha | 15,730 | 66.3 | 33.7 | 0.056 | -0.261 | 11,232 | 64.3 | 1,478 | 70.5 | 2,168 | 70.5 | 799 | 75.4 |
| *Flavoperla* sp. | Fsp | 15,796 | 68.3 | 31.7 | 0.037 | -0.262 | 11,220 | 67.1 | 1,492 | 70.7 | 2,197 | 70.9 | 866 | 72.8 |
| *Hemacroneuria ovalis* | Hov | 16,351 | 63.6 | 36.4 | 0.038 | -0.276 | 11,235 | 61.5 | 1,489 | 68.8 | 2,742 | 67.9 | 1,086 | 69.2 |
| *Hesperoperla pacifica* | Hpa | 15,666 | 61.9 | 38.1 | 0.112 | -0.329 | 11,235 | 60.4 | 1,476 | 67.5 | 2,131 | 67.1 | >413 | - |
| *Niponiella limbatella* | Nli | 15,924 | 63.1 | 36.9 | 0.063 | -0.295 | 11,226 | 60.9 | 1,479 | 69.3 | 2,163 | 67.7 | 1,052 | 69.8 |
| *Sinacroneuria dabieshana* | Sda | 15,752 | 67.3 | 32.7 | 0.059 | -0.304 | 11,223 | 65.8 | 1,481 | 68.8 | 2,177 | 69.8 | 851 | 77.5 |

**Table S3.** Features of the mitochondrial genome of *Hemacroneuria ovalis*.

| Gene | Direction | Location | Size | Anticodon | Codon  Start | Stop | Intergenic nucleotides |
| --- | --- | --- | --- | --- | --- | --- | --- |
| *trnI* | F | 1-67 | 67 | 29-31 GAT |  |  | 0 |
| *trnQ* | R | 65-133 | 69 | 101-103 TTG |  |  | -3 |
| *trnM* | F | 133-202 | 70 | 164-166 CAT |  |  | -1 |
| *ND2* | F | 203-1,237 | 1,035 |  | ATG | TAA | 0 |
| *trnW* | F | 1,236-1,302 | 67 | 1,267-1,269 TCA |  |  | -2 |
| *trnW* | F | 1,358-1,424 | 67 | 1,389-1,391 TCA |  |  | 55 |
| *trnW* | F | 1,480-1,546 | 67 | 1,511-1,513 TCA |  |  | 55 |
| *trnW* | F | 1,602-1,669 | 68 | 1,633-1,635 TCA |  |  | 55 |
| *trnC* | R | 1,662-1,729 | 68 | 1,697-1,699 GCA |  |  | -8 |
| *trnY* | R | 1,729-1,793 | 65 | 1,760-1,762 GTA |  |  | -1 |
| *COI* | F | 1,786-3,330 | 1,545 |  | ATT | TAA | -8 |
| *trnL2* | F | 3,326-3,391 | 66 | 3,355-3,357 TAA |  |  | -5 |
| *COII* | F | 3,399-4,086 | 688 |  | ATG | T | 7 |
| *trnK* | F | 4,087-4,157 | 71 | 4,117-4,119 CTT |  |  | 0 |
| *trnD* | F | 4,158-4,225 | 68 | 4,187-4,189 GTC |  |  | 0 |
| *ATP8* | F | 4,226-4,387 | 162 |  | ATC | TAA | 0 |
| *ATP6* | F | 4,381-5,058 | 678 |  | ATG | TAA | -7 |
| *COIII* | F | 5,092-5,878 | 787 |  | ATG | T | 33 |
| *trnG* | F | 5,879-5,945 | 67 | 5,909-5,911 TCC |  |  | 0 |
| *ND3* | F | 5,946-6,299 | 354 |  | ATC | TAG | 0 |
| *trnA* | F | 6,298-6,364 | 67 | 6,327-6,329 TGC |  |  | -2 |
| *trnR* | F | 6,364-6,428 | 65 | 6,394-6,396 TCG |  |  | -1 |
| *trnN* | F | 6,428-6,494 | 67 | 6,459-6,461 GTT |  |  | -1 |
| *trnS1* | F | 6,495-6,561 | 67 | 6,520-6,522GCT |  |  | 0 |
| *trnE* | F | 6,562-6,628 | 67 | 6,592-6,594 TTC |  |  | 0 |
| *trnF* | R | 6,630-6,695 | 66 | 6,662-6,664 TTC |  |  | 1 |
| *ND5* | R | 6,695-8,431 | 1,737 |  | ATG | TAG | -1 |
| *trnH* | R | 8,433-8,499 | 67 | 8,464-8,466 GTG |  |  | 1 |
| *ND4* | R | 8,500-9,840 | 1,341 |  | ATG | TAG | 0 |
| *ND4L* | R | 9,834-10,130 | 297 |  | ATG | TAA | -7 |
| *trnT* | F | 10,133-10,201 | 69 | 10,163-10,165 TGT |  |  | 2 |
| *trnP* | R | 10,203-10,269 | 67 | 10,236-10,238 TGG |  |  | 1 |
| *ND6* | F | 10,271-10,795 | 525 |  | ATT | TAA | 1 |
| *CytB* | F | 10,795-11,931 | 1,137 |  | ATG | TAG | -1 |
| *trnS2* | F | 11,930-11,999 | 70 | 11,961-11,963 TGA |  |  | -2 |
| *ND1* | R | 12,016-12,966 | 951 |  | TTG | TAG | 16 |
| *trnL1* | R | 12,968-13,033 | 66 | 13,002-13,004 TAG |  |  | 1 |
| *lrRNA* | R | 13,034-14,371 | 1,338 |  |  |  | 0 |
| *trnV* | R | 14,372-14,443 | 72 | 14,408-14,410 TAC |  |  | 0 |
| *srRNA* | R | 14,444-15,265 | 822 |  |  |  | 0 |
| CR |  | 15,266-16,351 | 1,086 |  |  |  | 0 |

**Table S4.** Features of the mitochondrial genome of *Hesperoperla pacifica*.

| Gene | Direction | Location | Size | Anticodon | Codon  Start | Stop | Intergenic nucleotides |
| --- | --- | --- | --- | --- | --- | --- | --- |
| *trnI* | F | 1-66 | 66 | 30-32 GAT |  |  | 0 |
| *trnQ* | R | 127-195 | 69 | 163-165TTG |  |  | 60 |
| *trnM* | F | 204-269 | 66 | 234-236CAT |  |  | 8 |
| *ND2* | F | 270-1,304 | 1,035 |  | ATG | TAA | 0 |
| *trnW* | F | 1,303-1,368 | 66 | 1,333-1,335TCA |  |  | -2 |
| *trnC* | R | 1,361-1,429 | 69 | 1,397-1,399GCA |  |  | -8 |
| *trnY* | R | 1,491-1,557 | 67 | 1,523-1,525 GTA |  |  | 61 |
| *COI* | F | 1,550-3,094 | 1,545 |  | ATC | TAA | -8 |
| *trnL2* | F | 3,090-3,154 | 65 | 3,119-3,121 TAA |  |  | -5 |
| *COII* | F | 3,160-3,847 | 688 |  | ATG | T- | 5 |
| *trnK* | F | 3,848-3,918 | 71 | 3,878-3,880 CTT |  |  | 0 |
| *trnD* | F | 3,918-3,984 | 67 | 3,946-3,948 GTC |  |  | -1 |
| *ATP8* | F | 3,985-4,146 | 162 |  | ATA | TAA | 0 |
| *ATP6* | F | 4,140-4,817 | 678 |  | ATG | TAA | -7 |
| *COIII* | F | 4,830-5,618 | 789 |  | ATG | TAA | 12 |
| *trnG* | F | 5,618-5,681 | 64 | 5,647-5,649 TCC |  |  | -1 |
| *ND3* | F | 5,682-6,035 | 354 |  | ATT | TAA | 0 |
| *trnR* | F | 6,094-6,158 | 65 | 6,123-6,125 TCG |  |  | 58 |
| *trnN* | F | 6,158-6,223 | 66 | 6,188-6,190 GTT |  |  | -1 |
| *trnS1* | F | 6,224-6,290 | 67 | 6,249-6,251 GCT |  |  | 0 |
| *trnE* | F | 6,291-6,356 | 66 | 6,323-6,325 TTC |  |  | 0 |
| *trnA* | F | 6,454-6,519 | 66 | 6,483-6,485 TGC |  |  | 97 |
| *trnF* | R | 6,648-6,712 | 65 | 6,680-6,682 GAA |  |  | 128 |
| *ND5* | R | 6,713-8,447 | 1,735 |  | GTG | T- | 0 |
| *trnH* | R | 8,448-8,516 | 69 | 8,483-8,485 GTG |  |  | 0 |
| *ND4* | R | 8,516-9,856 | 1,341 |  | ATG | TAA | -1 |
| *ND4L* | R | 9,850-10,146 | 297 |  | ATG | TAA | -7 |
| *trnT* | F | 10,149-10,215 | 67 | 10,179-10,181 TGT |  |  | 2 |
| *trnP* | R | 10,217-10,282 | 66 | 10,250-10,252 TGG |  |  | 1 |
| *ND6* | F | 10,284-10,808 | 525 |  | ATT | TAA | 1 |
| *CytB* | F | 10,808-11,944 | 1,137 |  | ATG | TAG | -1 |
| *trnS2* | F | 11,943-12,012 | 70 | 11,974-11,976 TGA |  |  | -2 |
| *ND1* | R | 12,032-12,982 | 951 |  | TTG | TAG | 19 |
| *trnL1* | R | 12,984-13,050 | 67 | 13,019-13,021 TAG |  |  | 1 |
| *lrRNA* | R | 13,051-14,381 | 1,331 |  |  |  | 0 |
| *trnV* | R | 14,382-14,453 | 72 | 14,418-14,4420 TAC |  |  | 0 |
| *srRNA* | R | 14,454-15,253 | 800 |  |  |  | 0 |
| CR |  | 15,254-15,666 | >413 |  |  |  | 0 |

**Table S5.** Codon number and Relative synonymous codon usage (RSCU) in the *Hemacroneuria ovalis* (left) and *Hesperoperla pacifica* (right) mitochondrial PCGs

| Amino acid | Codon | N | RSCU | N+ | RSCU | N- | RSCU |
| --- | --- | --- | --- | --- | --- | --- | --- |
| Phe | UUU(F) | 213/183 | 1.35/1.19 | 101/66 | 1.07/0.75 | 112/117 | 1.78/1.77 |
|  | UUC(F) | 102/125 | 0.65/0.81 | 88/110 | 0.93/1.25 | 14/15 | 0.22/0.23 |
| Leu | UUA(L) | 220/179 | 2.00/1.68 | 113/80 | 1.82/1.30 | 107/99 | 2.24/2.21 |
|  | UUG(L) | 142/131 | 1.29/1.23 | 32/9 | 0.51/0.15 | 110/122 | 2.31/2.72 |
|  | CUU(L) | 112/85 | 1.02/0.80 | 79/64 | 1.27/1.04 | 33/21 | 0.69/0.47 |
|  | CUC(L) | 72/71 | 0.66/0.67 | 67/68 | 1.08/1.11 | 5/3 | 0.10/0.07 |
|  | CUA(L) | 71/125 | 0.65/1.18 | 58/118 | 0.93/1.92 | 13/7 | 0.27/0.16 |
|  | CUG(L) | 42/47 | 0.38/0.44 | 24/30 | 0.39/0.49 | 18/17 | 0.38/0.38 |
| Ile | AUU(I) | 199/163 | 1.44/1.19 | 129/101 | 1.29/0.99 | 70/62 | 1.84/1.77 |
|  | AUC(I) | 77/111 | 0.56/0.81 | 71/103 | 0.71/1.01 | 6/8 | 0.16/0.23 |
| Met | AUA(M) | 110/116 | 1.36/1.22 | 68/75 | 1.56/1.56 | 42/41 | 1.12/0.87 |
|  | AUG(M) | 52/74 | 0.64/0.78 | 19/21 | 0.44/0.44 | 33/53 | 0.88/1.13 |
| Val | GUU(V) | 105/81 | 1.60/1.27 | 52/29 | 1.52/0.82 | 53/52 | 1.68/1.84 |
|  | GUC(V) | 40/51 | 0.61/0.80 | 28/47 | 0.82/1.32 | 12/4 | 0.38/0.14 |
|  | GUA(V) | 63/78 | 0.96/1.22 | 39/60 | 1.14/1.69 | 24/18 | 0.76/0.64 |
|  | GUG(V) | 55/45 | 0.84/0.71 | 18/6 | 0.53/0.17 | 37/39 | 1.17/1.38 |
| Ser | UCU(S) | 78/81 | 1.89/1.91 | 44/35 | 1.81/1.47 | 34/46 | 2.00/2.49 |
|  | UCC(S) | 39/39 | 0.95/0.92 | 32/37 | 1.32/1.55 | 7/2 | 0.41/0.11 |
|  | UCA(S) | 65/67 | 1.58/1.58 | 51/51 | 2.10/2.14 | 14/16 | 0.82/0.86 |
|  | UCG(S) | 21/20 | 0.51/0.47 | 5/6 | 0.21/0.25 | 16/14 | 0.94/0.76 |
| Pro | CCU(P) | 67/53 | 1.71/1.37 | 47/27 | 1.62/0.94 | 20/26 | 1.95/2.60 |
|  | CCC(P) | 51/44 | 1.30/1.14 | 44/42 | 1.52/1.46 | 7/2 | 0.68/0.20 |
|  | CCA(P) | 26/42 | 0.66/1.08 | 18/37 | 0.62/1.29 | 8/5 | 0.78/0.50 |
|  | CCG(P) | 13/16 | 0.33/0.41 | 7/9 | 0.24/0.31 | 6/7 | 0.59/0.70 |
| Thr | ACU(T) | 83/69 | 1.61/1.28 | 60/43 | 1.50/1.06 | 23/26 | 2.00/2.00 |
|  | ACC(T) | 58/71 | 1.13/1.32 | 52/65 | 1.30/1.60 | 6/6 | 0.52/0.46 |
|  | ACA(T) | 56/55 | 1.09/1.02 | 44/45 | 1.10/1.10 | 12/10 | 1.04/0.77 |
|  | ACG(T) | 9/20 | 0.17/0.37 | 4/10 | 0.10/0.25 | 5/10 | 0.43/0.77 |
| Ala | GCU(A) | 89/109 | 1.60/1.91 | 60/55 | 1.61/1.43 | 29/54 | 1.59/2.92 |
|  | GCC(A) | 68/72 | 1.23/1.26 | 58/68 | 1.56/1.77 | 10/4 | 0.55/0.22 |
|  | GCA(A) | 43/34 | 0.77/0.60 | 28/29 | 0.75/0.75 | 15/5 | 0.82/0.27 |
|  | GCG(A) | 22/13 | 0.40/0.23 | 3/2 | 0.08/0.05 | 19/11 | 1.04/0.59 |
| Tyr | UAU(Y) | 89/67 | 1.24/0.9 | 31/11 | 0.81/0.29 | 58/56 | 1.73/1.51 |
|  | UAC(Y) | 55/82 | 0.76/1.1 | 46/64 | 1.19/1.71 | 9/18 | 0.27/0.49 |
| His | CAU(H) | 34/26 | 0.86/0.63 | 24/10 | 0.75/0.31 | 10/16 | 1.33/1.78 |
|  | CAC(H) | 45/56 | 1.14/1.37 | 40/54 | 1.25/1.69 | 5/2 | 0.67/0.22 |
| Gln | CAA(Q) | 66/59 | 1.61/1.48 | 46/51 | 1.77/1.85 | 20/8 | 1.33/0.64 |
|  | CAG(Q) | 16/21 | 0.39/0.53 | 6/4 | 0.23/0.15 | 10/17 | 0.67/1.36 |
| Asn | AAU(N) | 102/75 | 1.33/1.09 | 56/34 | 1.06/0.73 | 46/41 | 1.96/1.86 |
|  | AAC(N) | 51/62 | 0.67/0.91 | 50/59 | 0.94/1.27 | 1/3 | 0.04/0.14 |
| Lys | AAA(K) | 44/38 | 1.24/1.09 | 32/31 | 1.68/1.59 | 12/7 | 0.73/0.45 |
|  | AAG(K) | 27/32 | 0.76/0.91 | 6/8 | 0.32/0.41 | 21/24 | 1.27/1.55 |
| Asp | GAU(D) | 47/42 | 1.25/1.11 | 29/20 | 1.07/0.77 | 18/22 | 1.71/1.83 |
|  | GAC(D) | 28/34 | 0.75/0.89 | 25/32 | 0.93/1.23 | 3/2 | 0.29/0.17 |
| Glu | GAA(E) | 53/51 | 1.28/1.24 | 38/45 | 1.69/1.88 | 15/6 | 0.79/0.35 |
|  | GAG(E) | 30/31 | 0.72/0.76 | 7/3 | 0.31/0.13 | 23/28 | 1.21/1.65 |
| Cys | UGU(C) | 28/20 | 1.51/1.03 | 4/1 | 1.00/0.20 | 24/19 | 1.66/1.31 |
|  | UGC(C) | 9/19 | 0.49/0.97 | 4/9 | 1.00/1.80 | 5/10 | 0.34/0.69 |
| Trp | UGA(W) | 65/75 | 1.21/1.46 | 52/59 | 1.51/1.76 | 13/16 | 0.68/0.89 |
|  | UGG(W) | 42/28 | 0.79/0.54 | 17/8 | 0.49/0.24 | 25/20 | 1.32/1.11 |
| Arg | CGU(R) | 10/18 | 0.63/1.16 | 8/5 | 0.84/0.51 | 2/13 | 0.32/2.26 |
|  | CGC(R) | 13/6 | 0.83/0.39 | 10/4 | 1.05/0.41 | 3/2 | 0.48/0.35 |
|  | CGA(R) | 19/29 | 1.21/1.87 | 14/26 | 1.47/2.67 | 5/3 | 0.80/0.52 |
|  | CGG(R) | 21/9 | 1.33/0.58 | 6/4 | 0.63/0.41 | 15/5 | 2.40/0.87 |
| Ser(s) | AGU(S) | 37/35 | 0.90/0.83 | 17/9 | 0.7/0.38 | 20/26 | 1.18/1.41 |
|  | AGC(S) | 27/24 | 0.65/0.57 | 19/18 | 0.78/0.75 | 8/6 | 0.47/0.32 |
|  | AGA(S) | 60/71 | 1.45/1.68 | 25/35 | 1.03/1.47 | 35/36 | 2.06/1.95 |
|  | AGG(S) | 3/2 | 0.07/0.05 | 1/0 | 0.04/0 | 2/2 | 0.12/0.11 |
| Gly | GGU(G) | 55/70 | 0.88/1.11 | 33/25 | 0.94/0.69 | 22/45 | 0.80/1.67 |
|  | GGC(G) | 25/34 | 0.40/0.54 | 19/23 | 0.54/0.64 | 6/11 | 0.22/0.41 |
|  | GGA(G) | 49/86 | 0.78/1.37 | 33/73 | 0.94/2.03 | 16/13 | 0.58/0.48 |
|  | GGG(G) | 121/62 | 1.94/0.98 | 55/23 | 1.57/0.64 | 66/39 | 2.40/1.44 |
